# Diagnostic accuracy of clinical signs and symptoms of COVID-19: A systematic review and meta-analysis to investigate the different estimates in a different stage of the pandemic outbreak

**DOI:** 10.7189/jogh.13.06026

**Published:** 2023-07-14

**Authors:** Kuan-Fu Chen, Tsai-Wei Feng, Chin-Chieh Wu, Ismaeel Yunusa, Su-Hsun Liu, Chun-Fu Yeh, Shih-Tsung Han, Chih-Yang Mao, Dasari Harika, Richard Rothman, Andrew Pekosz

**Affiliations:** 1 Department of Emergency Medicine, Chang Gung Memorial Hospital, Linkou, Taiwan; 2 Clinical Informatics and Medical Statistics Research Center, Chang Gung University, Taoyuan, Taiwan; 3 Department of Emergency Medicine, Chang Gung Memorial Hospital, Keelung, Taiwan; 4 Department of Clinical Pharmacy and Outcomes Sciences, University of South Carolina College of Pharmacy, Columbia, South Carolina, USA; 5 Harvard T.H Chan School of Public Health, Boston, Massachusetts, USA; 6 Health Management Center, Far Eastern Memorial Hospital, Taipei, Taiwan; 7 School of Medicine, International Health Program, National Yang Ming University, Taipei, Taiwan; 8 Division of Infectious Diseases, Department of Internal Medicine, Chang Gung Memorial Hospital, Linkou, Taiwan; 9 Graduate Institute of Clinical Medical Sciences, College of Medicine, Chang Gung University, Taoyuan, Taiwan; 10 Department of Emergency Medicine, Johns Hopkins University School of Medicine, Baltimore, Maryland, USA; 11 Department of Molecular Microbiology and Immunology, The Johns Hopkins Bloomberg School of Public Health, Baltimore, Maryland, USA

## Abstract

**Background:**

The coronavirus (COVID-19) pandemic caused enormous adverse socioeconomic impacts worldwide. Evidence suggests that the diagnostic accuracy of clinical features of COVID-19 may vary among different populations.

**Methods:**

We conducted a systematic review and meta-analysis of studies from PubMed, Embase, Cochrane Library, Google Scholar, and the WHO Global Health Library for studies evaluating the accuracy of clinical features to predict and prognosticate COVID-19. We used the National Institutes of Health Quality Assessment Tool to evaluate the risk of bias, and the random-effects approach to obtain pooled prevalence, sensitivity, specificity, and likelihood ratios.

**Results:**

Among the 189 included studies (53 659 patients), fever, cough, diarrhoea, dyspnoea, and fatigue were the most reported predictors. In the later stage of the pandemic, the sensitivity in predicting COVID-19 of fever and cough decreased, while the sensitivity of other symptoms, including sputum production, sore throat, myalgia, fatigue, dyspnoea, headache, and diarrhoea, increased. A combination of fever, cough, fatigue, hypertension, and diabetes mellitus increases the odds of having a COVID-19 diagnosis in patients with a positive test (positive likelihood ratio (PLR) = 3.06)) and decreases the odds in those with a negative test (negative likelihood ratio (NLR) = 0.59)). A combination of fever, cough, sputum production, myalgia, fatigue, and dyspnea had a PLR = 10.44 and an NLR = 0.16 in predicting severe COVID-19. Further updating the umbrella review (1092 studies, including 3 342 969 patients) revealed the different prevalence of symptoms in different stages of the pandemic.

**Conclusions:**

Understanding the possible different distributions of predictors is essential for screening for potential COVID-19 infection and severe outcomes. Understanding that the prevalence of symptoms may change with time is important to developing a prediction model.

Since the initial cases identified in Wuhan, China, the coronavirus disease (COVID-19) has spread worldwide at an alarming rate, changing our daily lives, causing millions of deaths, and resulting in an adverse socio-economic impact worldwide [1,2]. The World Health Organization (WHO) declared COVID-19 a global pandemic in March 2020; since then, countries across the world have been struggling to contain its spread [3].

With thousands of research articles already indexed in PubMed, the WHO database, and other bibliographic databases [4], data on the incidence, risk factors, case fatality rate, and clinical features of COVID-19 have been growing daily. Interestingly, the reported case fatality rates differ by clinical characteristics and by country [5-7]. As the number of COVID-19 cases remains high with newer variants emerging [8], its clinical features, epidemiological characteristics, and risk factors for mortality are still not completely understood [9-12]. Several systematic reviews and meta-analyses have characterised the prevalence, risk factors, and clinical presentations of COVID-19 [13-18], yet there is no comprehensive evidence synthesis of the predictability and prognostication of clinical features of COVID-19.

One essential measure to prevent COVID-19 from spreading is the timely recognition of infected patients. An increased understanding of the predictability of its clinical features could significantly improve public health interventions needed to contain the pandemic, and evidence suggest that the predictive value of the clinical features of COVID-19 could vary among different populations [16,19,20]. We conducted a systematic review and meta-analysis to provide an up-to-date qualitative and quantitative synthesis of evidence of the epidemiology, clinical characteristics, laboratory data, and outcomes of patients with COVID-19 among different age groups, countries, and outbreak stages.

## METHODS

We performed the study following the Meta-Analysis Of Observational Studies in Epidemiology (MOOSE) [21] and the Preferred Reporting Items for Systematic Reviews and Meta-analysis (PRISMA) 2020 guidelines [22,23], registering the protocol in PROSPERO (CRD42020176289) [24].

### Search strategy

We searched PubMed, EMBASE, Cochrane Library, Google Scholar, and WHO Global Health Library using keywords related to COVID-19 for articles published between 1 December 2019 and 30 April 2020. Additionally, we used the snowball search approach and hand-searched the references of the included articles to ensure all relevant studies were included [25]. For updating the umbrella review, we searched PubMed and Scopus for articles published between 1 November 2019 and 31 August 2021 (see Table S1 in the **Online Supplementary Document** for detailed search strategy).

### Eligibility criteria

We included cohort, cross-sectional, case-control, and case-series studies reporting the risk factors, clinical features, and outcomes of patients with COVID-19 confirmed by a positive result of reverse-transcriptase polymerase-chain-reaction of SARS-CoV-2. We did not set language restrictions to avoid language bias [26]. We also included articles that reported outcomes of patients without restriction to clinical settings (inpatients, outpatients, or general population). We excluded studies with pregnant women and neonates (owing to many asymptomatic evaluations), non-peer reviewed studies, non-human studies, non-original studies, duplicates, reviews, book series, case reports, case series with fewer than four subjects, and studies with ambiguously described data (e.g. “fever or cough”).

### Primary and secondary outcomes

The primary outcomes were the diagnostic performance of symptoms, demographics, comorbidities, and laboratory data in predicting COVID-19 infection in different stages of the pandemic. The secondary outcomes were the performance of predictors in prognosticating severe COVID-19 infection (e.g. mortality, pneumonia, ICU hospitalization). Applying the PICO framework, the two outcomes could be expressed as follows:

**Diagnostic:**
P: Patients with suspected COVID-19 infection
I: Symptoms, demographics, comorbidities, and laboratory data
C: Confirmed COVID-19 infection
O: Performance in predicting COVID-19 infection

**Prognostic**
P: Patients with confirmed COVID-19 infection
I: Symptoms, demographics, comorbidities, and laboratory data
C: Severe COVID-19 infection (e.g. mortality, pneumonia, ICU hospitalisation)
O: Performance in prognosticating severe COVID-19 infection

### Data extraction

We extracted data on study characteristics (e.g. author, country, study design, date of a study conducted, numbers of participants), patient characteristics (e.g. age, gender, ethnicity, comorbidities), symptoms (e.g. degree of fever, cough), laboratory data (e.g. white blood cell (WBC) and lymphocyte counts) and adverse outcomes (e.g. severity, mortality, hospitalisation). If the studies lacked information, we contacted their authors for the relevant data. We transformed medians and interquartile ranges into means and standard deviations, and standard errors into standard deviations, following the methodology proposed by Wan et al. [27]. In studies with overlapping patient populations, we included the most up-to-date and complete data. Additionally, we examined published systematic reviews on COVID-19 to ensure no study was missed.

### Study quality assessment

We used the National Institute of Health in the USA (NIH) Quality Assessment Tool to assess the quality of studies [28] (Table S2 in the **Online Supplementary Document**). Two reviewers screened the titles and abstracts, followed by the full texts of the remaining articles, after which they performed the data extraction and quality assessment, resolving discrepancies through discussion with a senior reviewer (CKF).

### Data synthesis

The summary measures estimated in this meta-analysis were means, standard deviations, prevalence, and weighted mean differences (WMDs), depending on the outcome. We used the WMD to summarise continuous variables such as WBCs and lymphocyte counts between severe and non-severe COVID-19 infection. We used the random-effects models to pool estimates of the prevalence of symptoms with the maximum likelihood methods and variance stabilising transformation [29-31]. While addressing the challenge of pooling the proportion of a symptom in cases where it was not reported, four common approaches are suggested. The first is to exclude studies without data on the symptom, while the second is to include studies that reported a zero proportion for the symptom. The third approach is to assume a fixed value for missing data, while the fourth is to use a model-based imputation method. The implications of each approach must be considered and sensitivity analyses conducted to assess the robustness of the results. Furthermore, meta-analyses with missing data require careful consideration and should be performed by experienced researchers with expertise in this field. We adopted the first approach, since studies that miss certain symptoms in the early stage could indicate that our society has not thoroughly recognised the manifestation of COVID-19 infection. Additionally, some eligible studies had a prevalence of various predictors equal to zero; hence, we computed the pooled estimates using the Freeman-Tukey double arcsine transformation [32]. We then used the bivariate random-effect models in synthesising evidence of diagnostic accuracy [33]. We also used the hierarchical summary receiver operating characteristic (HSROC) models, as proposed by Rutter and Gatsonis [34], to calculate the pooled sensitivity and specificity, and the area under the summary receiver operating characteristic (AUHSROC) curve.

We used the Fagan nomogram [35-37] to illustrate how clinicians could apply predictors to their daily practice. A nomogram is a two-dimensional graphical tool used for estimating the post-test results given a specific pre-test probability and the likelihood ratio of the predictor. We based the nomogram on the combination of the likelihood ratios of a series of predictors obtained from the meta-analysis.

We further examined the statistical heterogeneity visually inspecting forest plots, performing Cochran’s Q test, and calculating the *I*^2^ statistics [38]. For the *I*^2^ statistic, we considered values of <25%, 25-50%, and 50-75% as low, moderate, and high heterogeneity, respectively [38,39]. We determined the statistical significance of heterogeneity using the Cochrane Q test at a *P* < 0.1.

### Publication bias

We examined potential publication bias by visually inspecting funnel plots using sample size as the measure of precision on the y-axis in dealing with extremely low or high prevalence, rank correlation test, and Egger's regression test [40].

### Sensitivity analyses

We conducted sensitivity analyses to examine how the estimates changed according to study quality, transformation methods, study designs (sub-classified into cohort, case-series, and case-control studies), country, stage of the outbreak (displayed by date), age of patients (categorised into adults (>18 years) and children), and threshold of fever. We sub-classified ethnicity into Chinese and Non-Chinese settings. We used the random-effects Q-test for heterogeneity evaluate the difference between subgroups. For between-study heterogeneity, we performed the outlier analysis to explore the source of heterogeneity that may have been caused by one or more studies with extreme effect sizes. We further performed multivariable meta-regression analyses with bivariate binomial mixed-effect models to explore the sources of heterogeneity.

We conducted all statistical analyses with the “meta” package for general meta-analysis, and “meta4diag”or HSROC” packages for diagnostic meta-analysis in R version 3.6.3. (R Foundation for Statistical Computing, Vienna, Austria) and Stata 15.1 (Stata Corporation, College Station, Texas, USA).

## RESULTS

### Study selection and characteristics of the included studies

**Figure 1:**
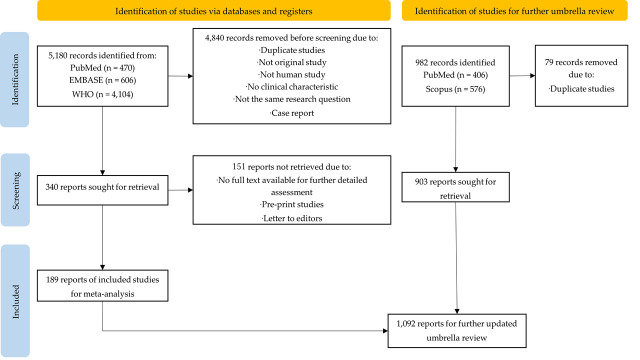
Flowchart of study identification, screening, inclusion, and exclusion in the systematic review, and further updating umbrella review. According to The PRISMA 2020 statement, the definitions of report, record, and study were as following: Report – a document supplying information about a particular study. Record – the title or abstract of a report indexed in a database or website. Study – an investigation, such as a clinical trial, that includes a defined group of participants, interventions, and outcomes.

We included 189 of 5180 reports retrieved by the initial search in the meta-analysis (**Figure 1**). Because of the ongoing pandemic, we further updated 982 reports from databases for umbrella review. After removing 79 duplicates, we finally included 1092 reports, and analysed the period effects on the clinical presentations (see search strategy in Table S1 in the **Online Supplementary Document**).

Most of the reports included in the initial systematic review were cohort studies (n = 129), followed by case series (n = 52) and case-control studies (n = 8). We included reports from 11 countries, and 171 studies reported data from China. The adults in the included studies had an average age of 50.4 years. The most reported symptoms were fever (n = 182), cough (n = 169), diarrhoea (n = 110), dyspnoea (n = 95), and fatigue (n = 94) (see Table S3 in the **Online Supplementary Document** for detailed study characteristics).

Because the COVID-19 pandemic continued, we further updated studies to better understand period effect. At this stage, most reports were from cohort studies (n = 712), followed by case series (n = 317), and case-control studies (n = 63).

### Distribution of symptoms of patients with COVID-19

We included variables such as mortality rates, the prevalence of severity of COVID-19 infection, fever, cough, sputum production, sore throat, myalgia, fatigue, dyspnoea, headache, nausea, and diarrhoea in the meta-analyses (Table S3 in the **Online Supplementary Document**).

**Figure 2:**
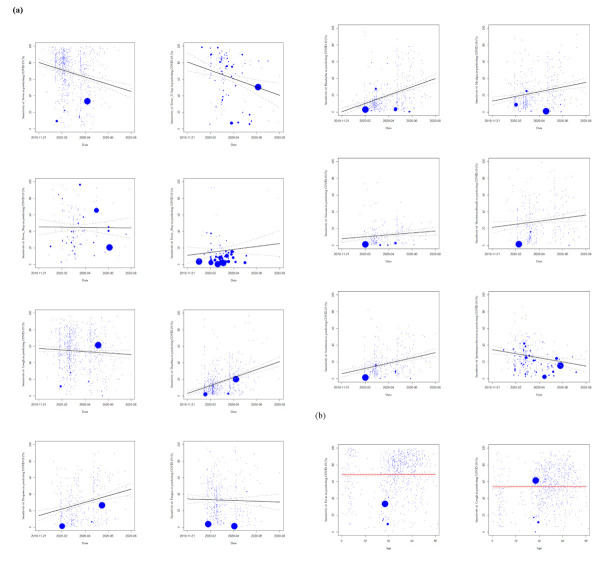
**Panel A.** Sensitivity of fever, cough, fatigue and dyspnoea in COVID-19 decreases with the stage of the outbreak. **Panel B.** Sensitivity of fever, and cough mildly increase with age in diagnosis. Other figures are presented in Figure S3 in the **Online Supplementary Document**.

After incorporating the updated data, fever (69%; 95% confidence interval (CI) = 68, 71) and cough (54%; 95% CI = 52, 56) were the two most common symptoms. In the later stage of the pandemic, the two symptoms decreased in sensitivity in predicting COVID-19. The sensitivity of other symptoms (sputum production, sore throat, myalgia, fatigue, dyspnoea, headache, and diarrhoea) increased (**Figure 2**).

**Figure 3:**
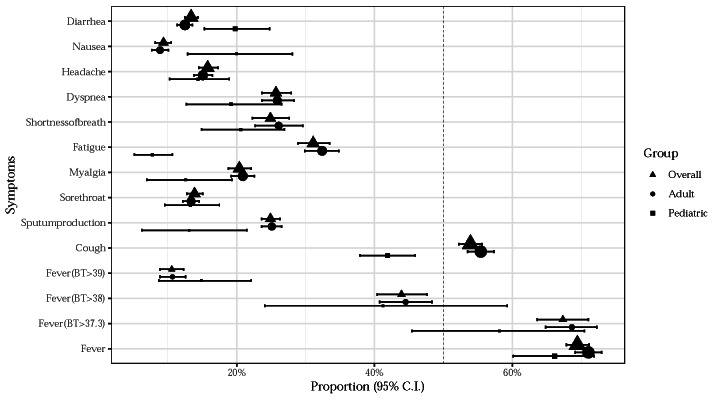
Forest plot for the proportion of symptoms and outcomes in patients with COVID-19 among the overall and different age groups. The definitions of the threshold for fever vary in different studies included. Fever of different thresholds (37.3°C, 38.0°C, 39.0°C) are displayed here.

The overall sensitivity of fever among COVID-19 cases reported in 1092 reports was 69% (95% CI = 68, 71) (**Figure 3**). The sensitivity of fever was lower among children and in non-Chinese studies (66% vs 71% and 62% vs 75%) (Figure S2b in the **Online Supplementary Document**). Cough (the second most common reported symptom) had a prevalence of 54% (95% CI = 52, 56) and was less common among children (42% vs 55%) (**Figure 3**).

**Figure 4:**
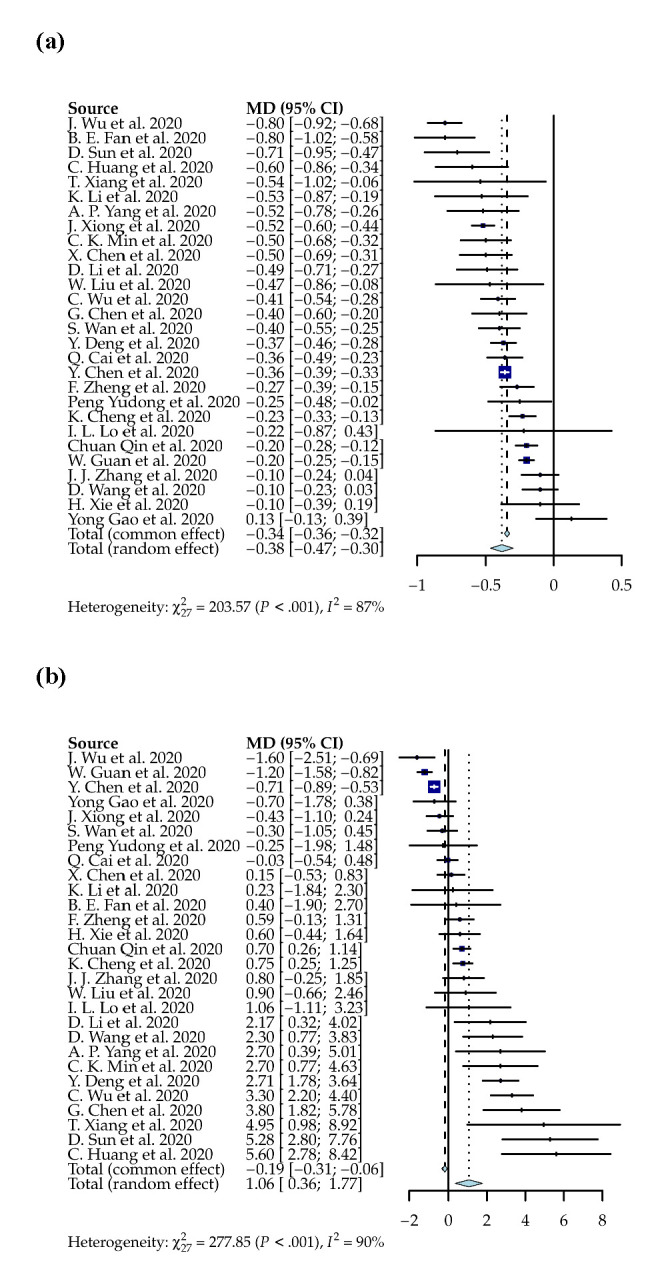
**Panel A.** Weighted mean differences of white blood cell counts between severe and non-severe patients with COVID-19. **Panel B.** Weighted mean differences of lymphocyte count between severe and non-severe patients with COVID-19.

The overall prevalence of severe cases among patients with COVID-19 reported in 27 studies was 31% (95% CI = 25, 37). The most present symptoms of severe cases were fever (89%; 95% CI = 83, 92), followed by cough (71%; 95% CI = 63, 78). The severe cases had a significantly higher WBC counts (WMD = 1.06 × 10^9^/L; 95% CI = 0.36, 1.77; *P* < 0.01) and lower lymphocyte counts (-0.38 × 10^9^/L; 95% CI = -0.47, -0.3; *P* < 0.01), while the pooled WBCs for severe and non-severe cases were 5.89 × 10^9^/L (95% CI = 5.45, 6.33) and 5.00 x10^9^/L (95% CI = 4.75, 5.26), respectively, and the pooled lymphocyte counts for severe and non-severe cases were 0.78 × 10^9^/L (95% CI = 0.72, 0.84) and 1.16 × 10^9^/L (95% CI = 1.10, 1.23), respectively (**Figure 4**).

### Diagnostic and prognostic performance analysis

**Figure 5:**
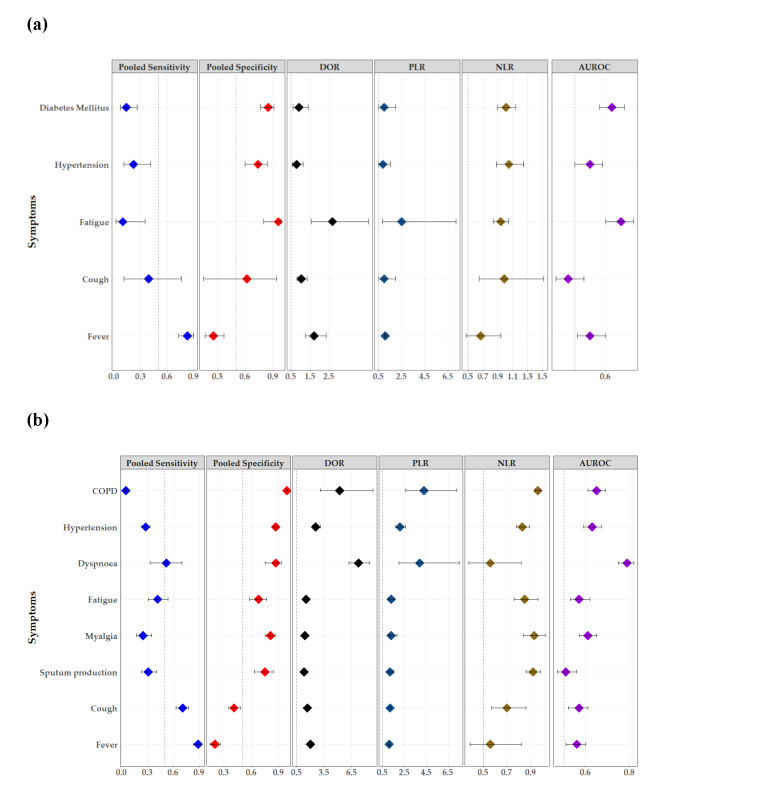
Forest plots. **Panel A.** Performance of predictors in detecting COVID-19 infection. **Panel B.** Performance of predictors in prognosticating severe COVID-19 infection. DOR – diagnostic odds ratio, PLR – positive likelihood ratio, NLR – negative likelihood ratio, AUROC – area under the receiver operating characteristic. Forest plots.

The studies for diagnostic and prognostic performance analysis only included adult patients. The most sensitive symptom in diagnostic of patients with COVID-19 was fever, with a fair pooled sensitivity of 83% (95% CI = 73, 90), but a poor overall performance (AUHSROC = 0.55; 95% CI = 0.51, 0.60) (**Figure 5****,** panel A). The most specific predictor in predicting COVID -19 was fatigue (96%; 95% CI = 80, 99), followed by diabetes mellitus (85%; 95% CI = 77, 91), and hypertension (74%; 95% CI = 60, 84) (**Figure 5****,** panel A). The overall PLR to predict COVID-19 of the combination of five predictors (fever, cough, fatigue, hypertension, and diabetes mellitus), was 3.06, whilst the overall NLR was 0.59.

Fever was also the most sensitive symptom in predicting severe COVID-19 cases (89%; 95% CI = 83, 92), followed by cough (71%; 95% CI = 63, 78) (**Figure 5****,** panel B). The most specific predictor of severe COVID-19 was chronic obstructive pulmonary disease (COPD) (99%; 95% CI = 98, 99), followed by diabetes mellitus (93%; 95% CI = 91, 95), hypertension (87%; 95% CI = 84, 89), and dyspnoea (87%; 95% CI = 75, 93) (**Figure 5****,** panel B). Moreover, we developed the nomogram to aid in predicting the risk of severe COVID-19 infection (Figure S1 in the **Online Supplementary Document**). The overall PLR in predicting severe COVID-19 of the combination of six symptoms (fever, cough, sputum production, myalgia, fatigue, and dyspnoea) was 10.44, while the overall NLR was 0.16.

### Adverse outcome assessment

The overall pooled mortality rate among the reported COVID-19 cases was 10% (95% CI = 6, 14) (Table S3 in the **Online Supplementary Document**). In sub-group analyses, mortality rates were lower among children, (0% vs 12%; *P* < 0.0001), in Chinese studies (9% vs 15%; *P* > 0.05) and in cohort studies compared to case series (9% vs 17%; *P* > 0.05), respectively (Table S3 in the **Online Supplementary Document**).

### Publication bias

We found significant publication bias by Egger's test in the following symptoms: fever, cough, sore throat, myalgia, fatigue, dyspnoea, headache, nausea, and diarrhoea (Table S3 in the **Online Supplementary Document**).

### Sensitivity analyses

Interestingly, we found sensitivity of fever was strongly correlated with the thresholds (75% for 37.3°C, 45% for 38°C, and 16% for 39°C) chosen based on clinical practice guidelines or previous studies which used them to define fever in adults. We found no significant difference among different transformation methods such as logit transformed (PLO), arcsine transformation (PAS), Freeman-Tukey double arcsine transform (PFT), and generalised linear mixed model (GLMM) (Table S4 in the **Online Supplementary Document**). However, we found substantial heterogeneity with *I*^2^>75% in mortality, the prevalence of severe COVID-19, and symptoms including fever, cough, sputum production, sore throat, myalgia, fatigue, dyspnoea, headache, nausea, and diarrhoea (Table S3 in the **Online Supplementary Document**). Some prevalence of the symptoms among patients with COVID-19 ranged widely among different study designs. In case-control studies, the prevalence of fever, cough, myalgia, fatigue, and nausea was found to be higher than in the cohort studies (Table S3 in the **Online Supplementary Document**). After removing 31 studies by outlier analysis, the pooled mortality rate decreased insignificantly from 10% to 9% (Egger's test = -2.375; *P* > 0.05) (Table S5 in the **Online Supplementary Document**). After including the studies retrieved from the updated search, we still found the sensitivity of fever was strongly correlated with the thresholds (66% for 37.3°C, 44% for 38°C, and 12% for 39°C). We found no statistically significant differences between the included case series studies and other studies.

### Meta-regression results

We identified the correlation between the sensitivity of symptoms like fever, cough, fatigue, and dyspnoea, and the potential confounders, such as the outbreak stage, age, and comorbidities.

The sensitivity of fever in COVID-19 cases decreased with the stage of the outbreak (**Figure 5****,** panel A). The threshold of 37.3°C is best correlated with the overall trend. Similarly, symptoms, such as cough, fatigue, and sputum production were negatively correlated with outbreak stage.

The sensitivity of fever, cough, and fatigue was positively correlated with age (**Figure 5****,** panel B). In contrast, diarrhoea, headache, and sore throat were negatively correlated with age.

### Quality assessment

Most studies lacked adequate justification of sample size and blinding of the outcome assessment (Figure S2 in the **Online Supplementary Document**). The other common risks of bias were the lack of confounder adjustment, prospective measurement of exposure, sample size justification, and blinding of the outcome assessment. In the subsequent sensitivity analysis, we found predictors such as fever, headache, and sore throat could be over-estimated in non-prospectively measured exposure and the lack of concurrent controls.

## DISCUSSION

To our knowledge, this is the largest meta-analysis to evaluate predictors for COVID-19 and severe COVID-19 infection, encompassing 1092 studies with 3 342 969 patients. In our quantitative synthesis, approximately one in five test-positive adults were not febrile, and we found that the sensitivity threshold of fever should be 37.3°C. We found a lower prevalence (i.e. sensitivity) of symptoms among patients with COVID-19 than in the earlier studies, and demonstrated that the stage of the outbreak is an important confounding factor. We also presented two nomograms (Figure S1 in the **Online Supplementary Document**) with satisfactory positive and negative likelihood ratios by combining symptoms to provide direction to the front-line clinicians in their daily practice confronting the COVID-19 pandemic.

We found lower sensitivity of symptoms among patients with COVID-19 than in the previous smaller-scale systematic reviews and meta-analyses [41,42]. We found that the most reported predictors were fever, cough, diarrhoea, dyspnoea, and fatigue, in line with other studies [43]. One systematic review and meta-analysis of seven studies indicated fever as the most prevalent clinical symptom (91.3%; 95% CI = 86, 97), followed by cough (67.7%; 95% CI 59, 76), fatigue (51.0%; 95% CI = 34, 68) and dyspnoea (30.4%; 95% CI = 21, 40) [41]. Similarly, two larger systematic reviews and meta-analyses of over twenty studies consisting of patients with more severe infections had similar findings [18,42].

Cochrane published an updated systematic review with 90 studies and conducted meta-analyses for 13 symptoms (fever, dyspnoea, cough, diarrhoea, sore throat, fatigue, rhinorrhoea, headache, anosmia or ageusia, ageusia, myalgia, chills/shivers) [10]. In its previous version, fever (53.8%; 95% CI = 35.0, 75.7) and cough (67.4%; 95% CI = 59.8, 74.1) were the most sensitive symptoms [11]. However, the updated study [10] found that cough was the only symptom with a sensitivity of over 50% (95% CI = 50.6, 72.9), while fever showed a decreased sensitivity of 37.6% (95% CI = 23.4, 54.3), indicating that outbreak stage affected sensitivity. The study also highlighted existing selection bias, because of selective and non-random inclusion, and identified spectrum bias.

However, a recent systematic review and meta-analysis [44] had 10% lower estimates than prior ones, which is in line with our findings. However, the authors did not examine the risk of bias in the included studies and they only used the dichotomized fever.

The different prevalence of fever among patients with COVID-19 infection might also be attributed to different thresholds used in the studies. We found that the prevalence of fever (ie, sensitivity) in patients with COVID-19 ranged from 67% for 37.3°C to 44% for 38°C. Since a thermal detector may the only measure used to screen potential patients with COVID-19 in many resource-limited settings, it is crucial that we understand the different thresholds and their related sensitivities.

We further explored the effect of different outbreak stages in view of these discrepancies. We found a lower sensitivity of fever and cough, fatigue, and sputum production among studies conducted at a later outbreak stage, possibly due to different patient selection, different definitions of the disease syndrome, and different resource use. Zhang et al. [45] proposed that the clinical features of patients with COVID-19 might differ in different outbreak stages . The insufficient understanding of the virus, the shortage of medical resources, and the spectrum of the patients reported might be the reasons that patients in different outbreak stages had different manifestations [46]. Zhang et al. [45] also indicated that fever and cough appeared at the early stage of the COVID-19 infection. Accordingly, clinicians should be attentive of the stage of the pandemic or epidemic in their practice locations, and the patient’s infection stage.

We also found a lower prevalence of fever, cough, fatigue, dyspnoea, sputum production, and severe cases among younger patient populations. In line with an updated meta-analysis [47], we found that around half of the paediatric patients with COVID-19 had a fever. It is purposed that children might have an under-developed immune system, which might inversely prevent the likelihood of severe infection induced by an over-reacting immune response [48].

In this study, we found the pooled mortality rate to be 10% and the prevalence of severe infection to be 31%. We also found fever as the most sensitive predictor, and COPD was the most specific predictor for severe COVID-19. These two predictors are not commonly reported in current literature, in which some other comorbidities such as hypertension, respiratory system disease, and cardiovascular disease are associated with severity [41,49]. However, our findings for COPD are consistent with the results of previous meta-analyses, which reported that COPD was the strongest comorbidity that predicted severe disease [16,17]. We also found that leukocytosis and lymphopenia were associated with severe COVID-19 along with other researchers [50,51]. We also provided pooled WBC and lymphocyte counts, along with their weighted mean difference between severe and non-severe patients with COVID-19 in 27 studies. This phenomenon is supported by the theory that the T lymphocyte cells (including CD4 and CD8 cells) might be killed by viruses such as influenza in severe cases and result in profound lymphopenia [52,53].

Many other confounders could be influencing the diagnostic and prognostic performance of the predictors in the included observational studies. One of the most important factors is the selection of study populations. As we indicated in our subgroup analyses, case-control studies tend to give overestimated prevalence of symptoms among patients with COVID-19. Second, age and many comorbidities could be influencing the performance of these predictors. According to our multivariable meta-regression results, the prevalence of diarrhoea, dyspnoea, myalgia, and headache among patients with COVID-19 increased slightly, while fever, cough, fatigue, diarrhoea, and nausea decreased slightly. Therefore, we caution the clinicians to consider different sensitivities in different patient populations.

We provided two nomograms (Figure S1 in the **Online Supplementary Document**) to assist front-line clinicians in predicting and prognosticating COVID-19 in their daily practice. Assuming the independence of these symptoms, patients with fever, cough, sputum production, sore throat, myalgia, and fatigue would be 3-fold more likely to have a positive COVID-19 infection, which could increase a presumed 10% pre-test probability to 25% post-test probability (**Figure 3**). Similarly, clinicians could use the combination of another six predictors, including fever, cough, sputum production, sore throat, myalgia, and fatigue to increase their post-test likelihood 10-fold from a presumed 10% pre-test probability to 54% post-test probability for severe COVID-19 infection.

We found that, due to the widespread administration of vaccines in the latter half of the pandemic, there has been a change in the presentation of COVID-19 symptoms among confirmed cases. In 2022, Tian et al. [54] conducted a systematic review and meta-analysis on the clinical characteristics and presentation of patients who received COVID-19 vaccinations vs those who did not. The results showed that the vaccinated group exhibited a significant reduction in certain clinical symptoms, such as fever and cough. Also, they found that vaccination could reduce the severity of disease (consistent with the study by Giuseppe et al. [55]) also found and more patients with asymptomatic infection in the vaccinated group.

We included case series in our study despite their limitations (e.g. lack of a control group, potential for selection bias), as they can provide valuable insights into the characteristics of COVID-19-infected patients. In the early stage of the pandemic outbreak, only case series were available to provide insight into predicting and prognosticating patients with suspected COVID-19 infection. Accordingly, we performed a sensitivity analysis to evaluate the potential impact and found no statistically significant differences between the case series and other studies.

Our study has several limitations. First, to maximize the sample size and deliver the most confident estimates of the predictors, we pooled different kinds of studies together. However, in the subsequent sensitivity analyses, we provided further detailed results for the clinicians to use. Second, at the beginning of the outbreak, patients included in these studies might have more severe disease manifestation, given the lack of medical resources, attention, and consistent screening and diagnostic criteria. Nevertheless, we also provided adjusted pooled sensitivity of these predictors in the meta-regression to minimise the influence of age, comorbidities, and outbreak stage. Third, although we attempted to evaluate the influence of the risk of bias in the subgroup analyses, we could not incorporate different risks of bias in different study designs and were only able to provide the potential influence in the sensitivity analyses. Fourth, few studies provided results from the control group, which might influence the precision of specificity in our study. Fifth, smell and taste dysfunction, or olfactory and gustatory dysfunctions, were discussed in different studies as an important symptom of COVID-19. Previous systematic reviews indicated that the prevalence of olfactory dysfunction and gustatory dysfunctions were 41.0% (95% CI = 28.5, 53.9) and 38.2% (95% CI = 24.0, 53.6), respectively [56]. However, most studies did not record them as they were not recognised during the early pandemic stages, so we did not have enough data for an accurate analysis. Sixth, while we converted the median to mean using a commonly employed method, we acknowledge that this approach is not perfect and may not accurately reflect the true mean in all instances. Seventh, not all symptoms were reported by all studies. We excluded studies without data on the symptoms, which might falsely inflate the prevalence of the symptoms. Lastly, the composite criteria of severe COVID-19 used in different studies might influence the predictor performance.

## CONCLUSIONS

In this systematic review and meta-analysis, approximately one in five test-positive adults were not febrile, and fever (followed by cough) was the most sensitive symptom in predicting the severity in confirmed COVID-19 cases, while COPD was the most specific predictor of severe COVID-19.

We found that outbreak stage and age impacted the prevalence of fever, cough and dyspnoea. Understanding the possible different distribution of predictors is essential to screening for potential COVID-19 infection and severe outcomes. The combination of symptoms could improve the pre-test probability before screening for potential infection and severe outcomes.

## Additional material


Online Supplementary Document
