# Supplementary material for: Diagnostic accuracy of clinical signs and symptoms of COVID-19: A systematic review and meta-analysis to investigate the different estimates in a different stage of the pandemic outbreak
Source: J Glob Health. 2023 Jul 14;13:06026. doi: 10.7189/jogh.13.06026 (PMC10344460; doi:10.7189/jogh.13.06026)
Supplement: Online Supplementary Document [file jogh-13-06026-s001.pdf]

Figure S1. Flow chart of the analytic steps in this study.

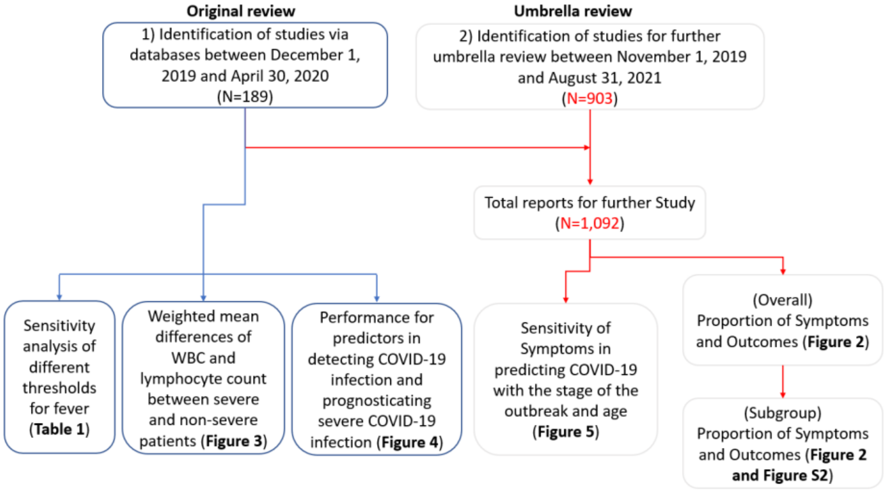

Figure S2. Missing pattern and proportions of extracted data from included studies.

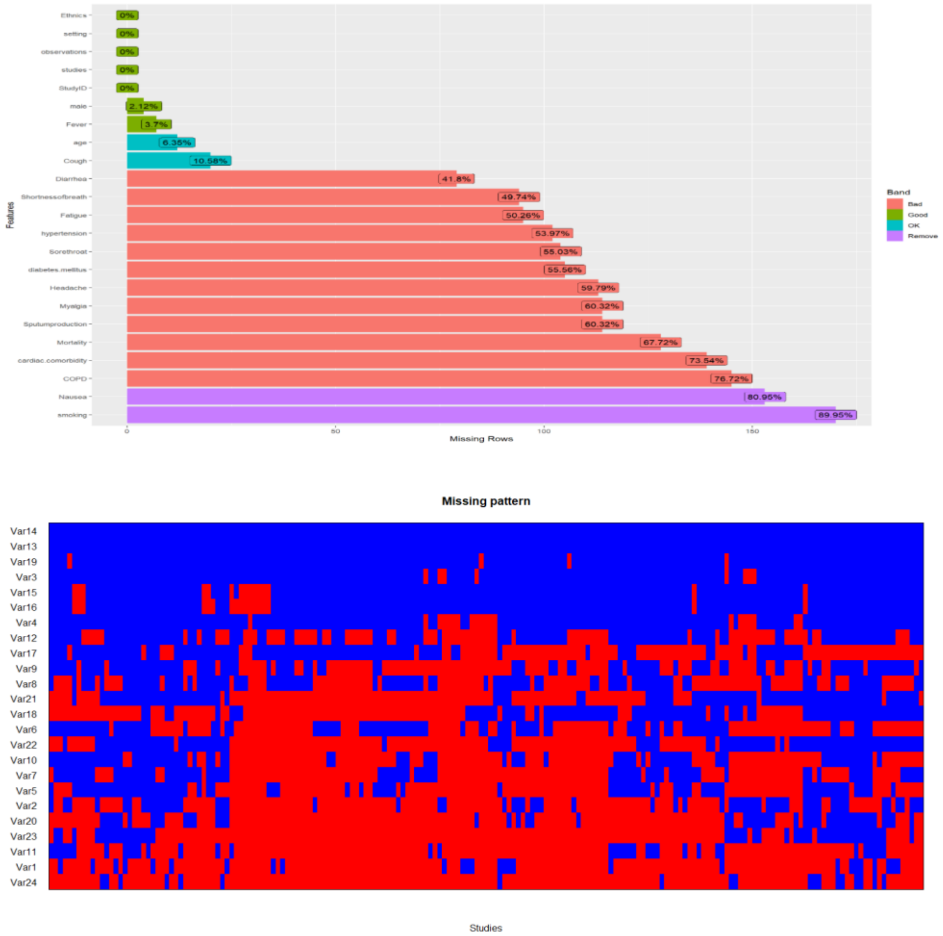

Note: Var1: Severity, Var2: Mortality, Var3: Fever, Var4: Cough, Var5: Sputum production, Var6: Sore throat, Var7: Myalgia, Var8: Fatigue, Var9: Dyspnea, Var10: Headache, Var11: Nausea, Var12: diarrhoea, Var13: Setting, Var14: Ethnics, Var15: Data_first, Var16: Data_last, Var17: age (mean), Var18: age (median), Var19: Gender, Var20: Cardiac comorbidity, Var21: Hypertension, Var22: Diabetes mellitus, Var23: COPD, Var24: Smoking

Figure S3a. Forest plot for the proportion of symptoms among different study design groups.

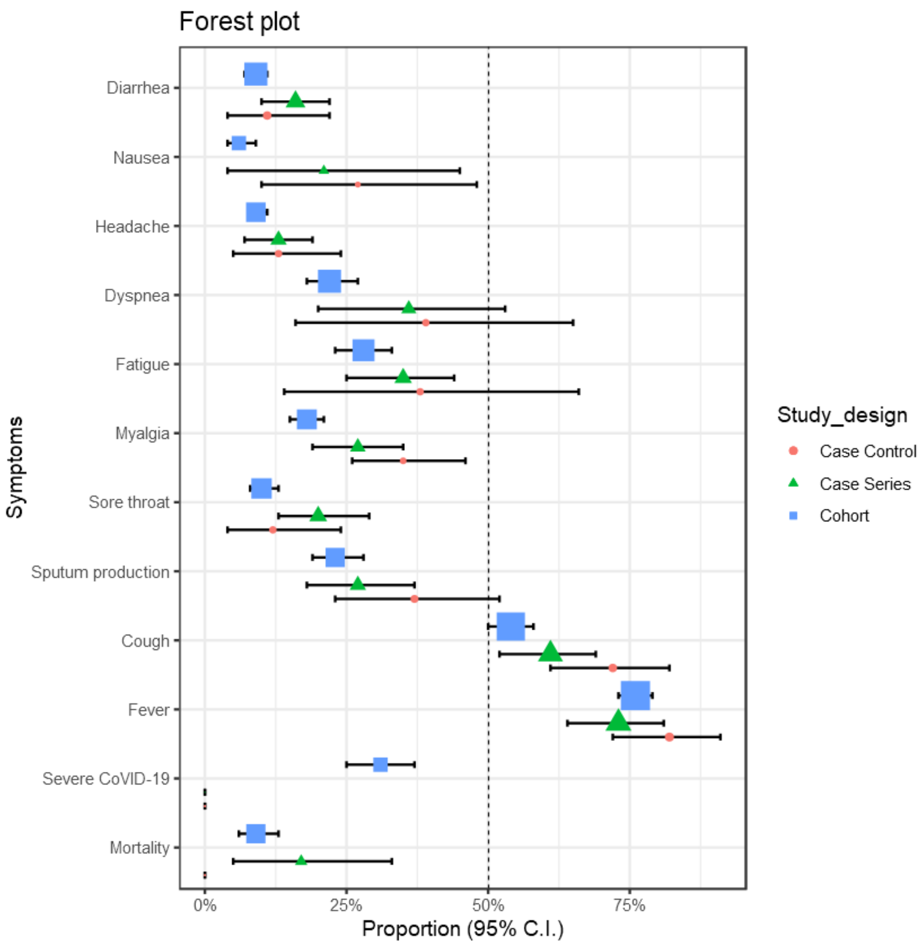

Figure S3b. Forest plot for the proportion of symptoms among different ethnicity groups.

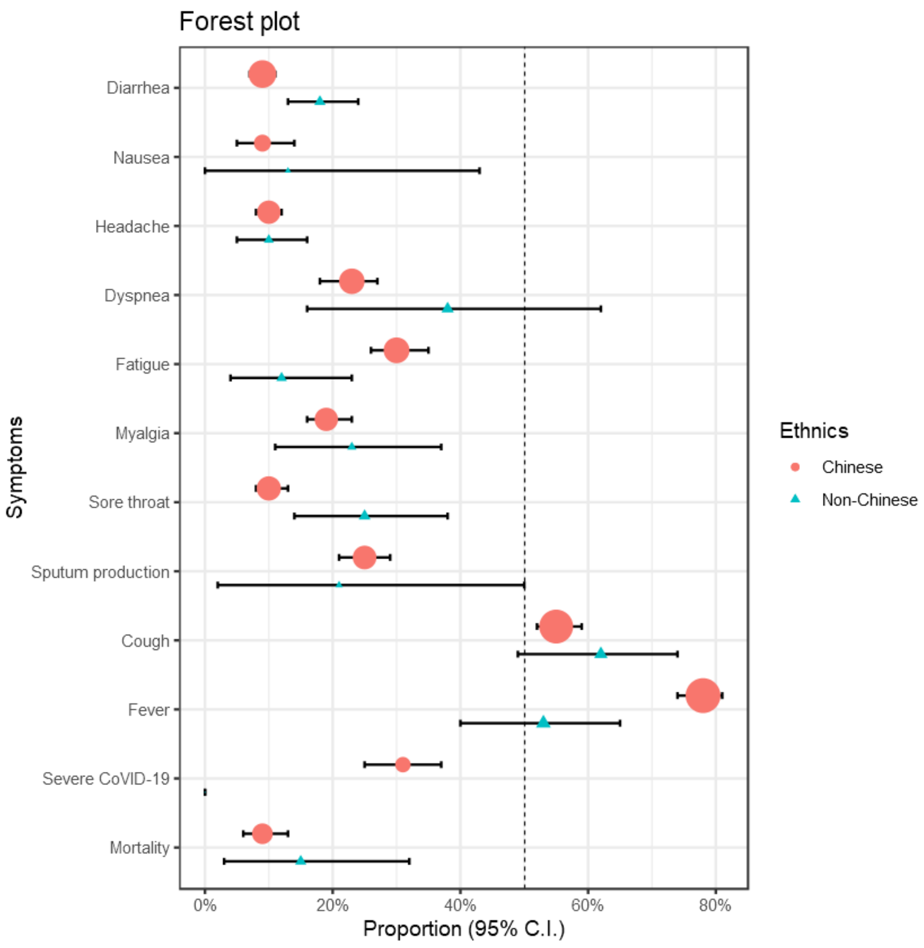

Figure S4a. Forest plot of an overall mean from included studies for WBCs.

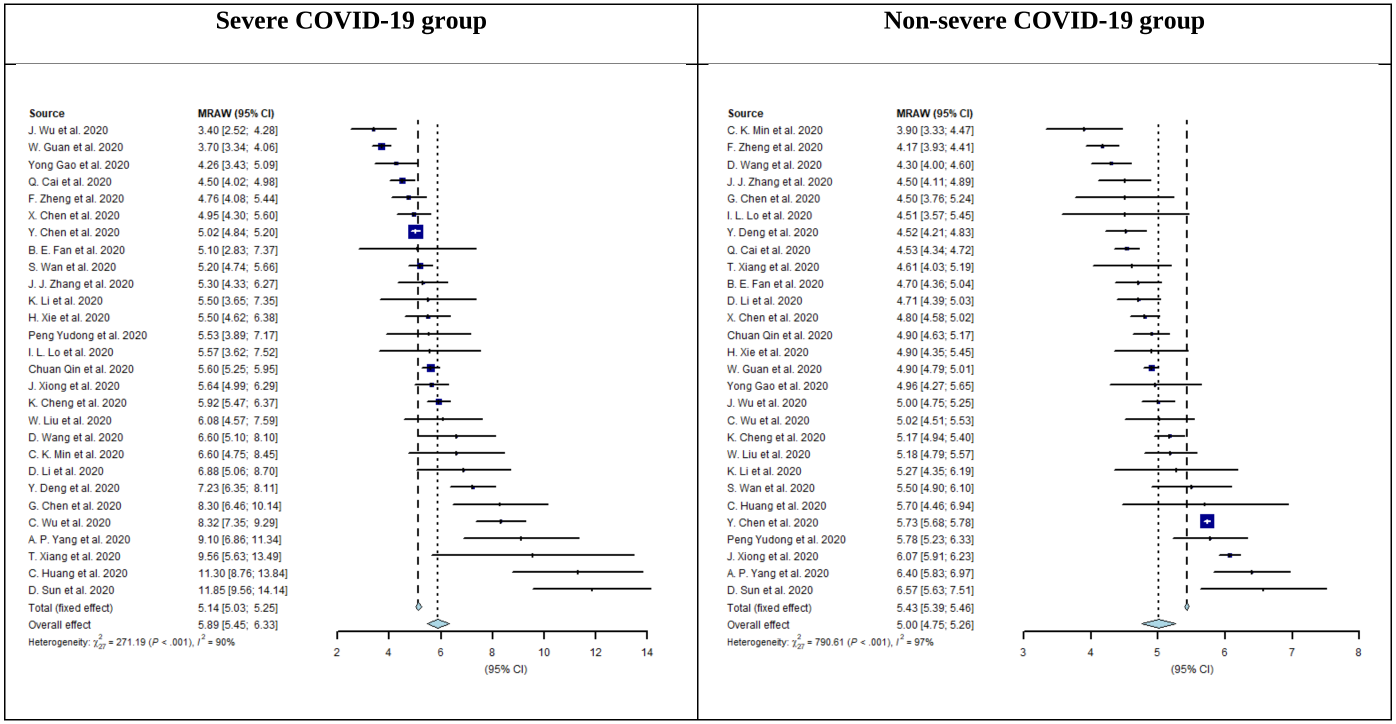

Figure S4b. Forest plot of an overall mean form included studies for lymphocyte counts.

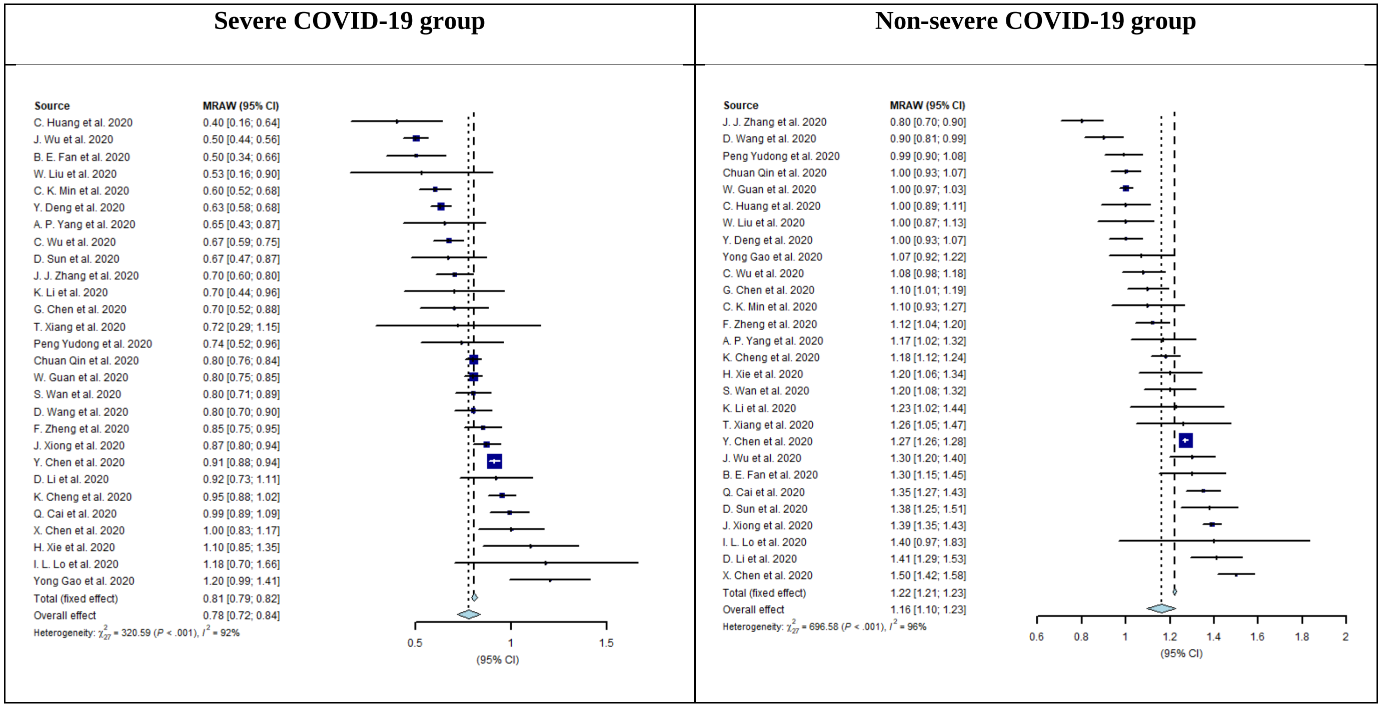

Figure S5a. Prevalence of fever, cough, fatigue, dyspnea of COVID-19 patients decrease with stage of the outbreak in.

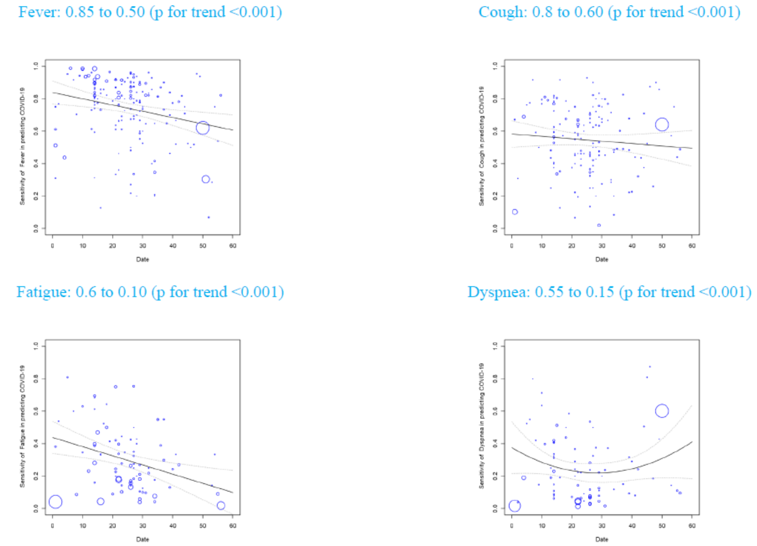

Figure S5b. *Prevalence of fever, cough, fatigue, dyspnea increase with age in diagnosis.*

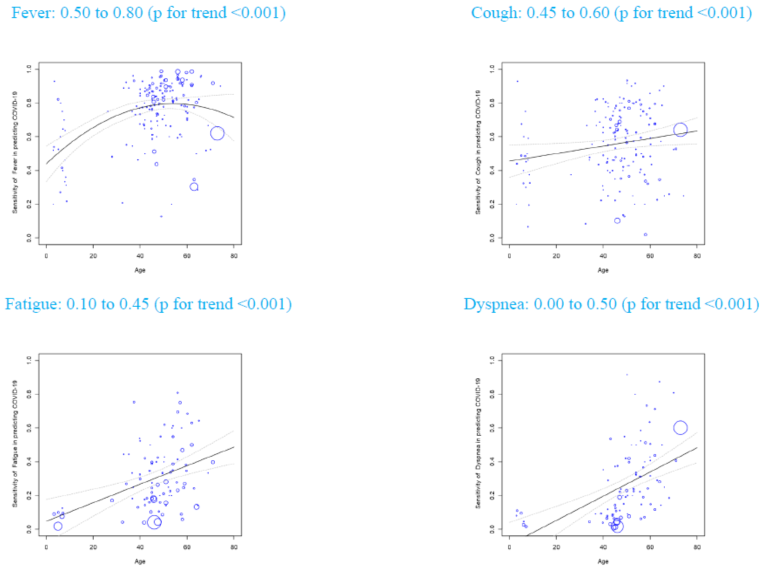

Figure S5c. Period effect in severe CoVID-19 group.

Fever

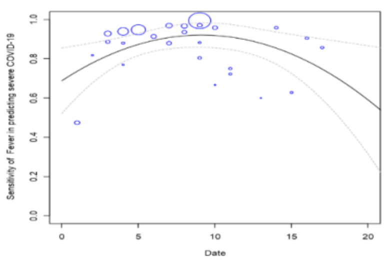

Cough

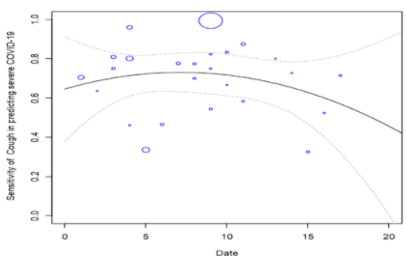

Sputum production

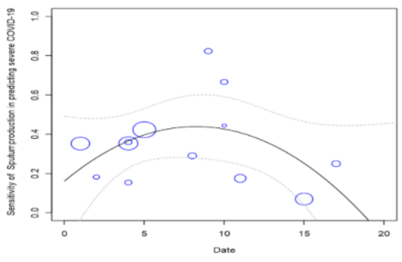

Sore throat

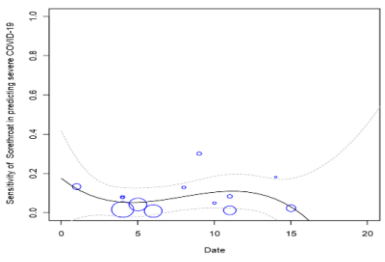

Myalgia

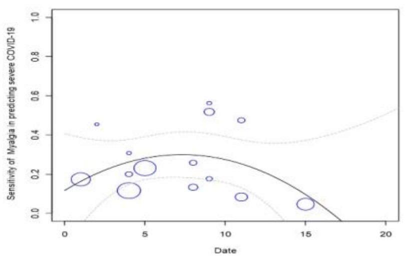

Fatigue

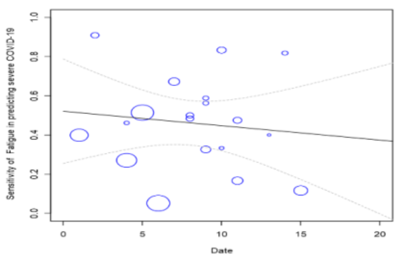

Note: The size of each circle has represented the inverse of the square root of effect size for each study. Date was represented the beginning enrolled day for each study (From 2019/12/1 to 2020/1/31).

Figure S6a. Graphical Display of quality assessment for cohort study.

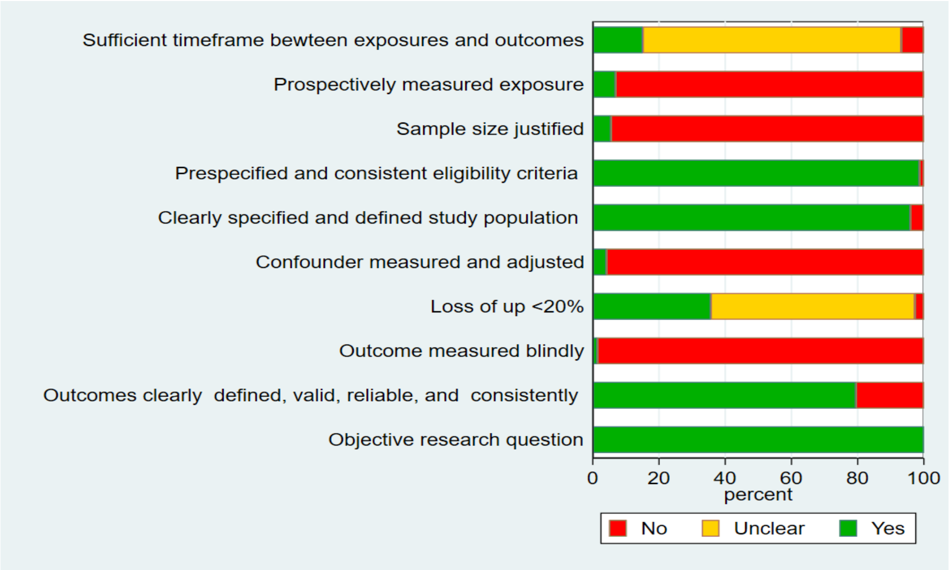

Figure S6b. Graphical Display of quality assessment for case series study.

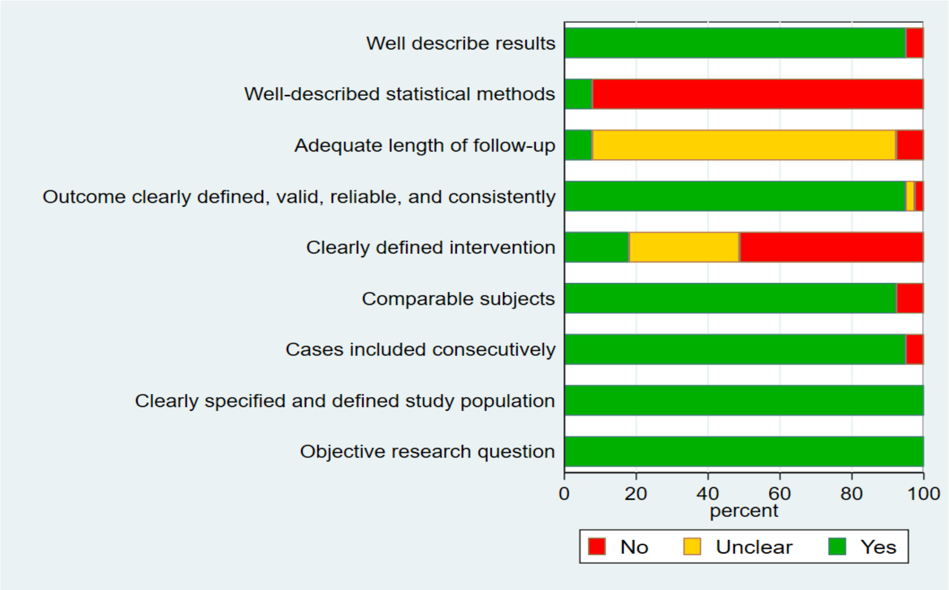

Figure S6c. Graphical Display of quality assessment for case control study.

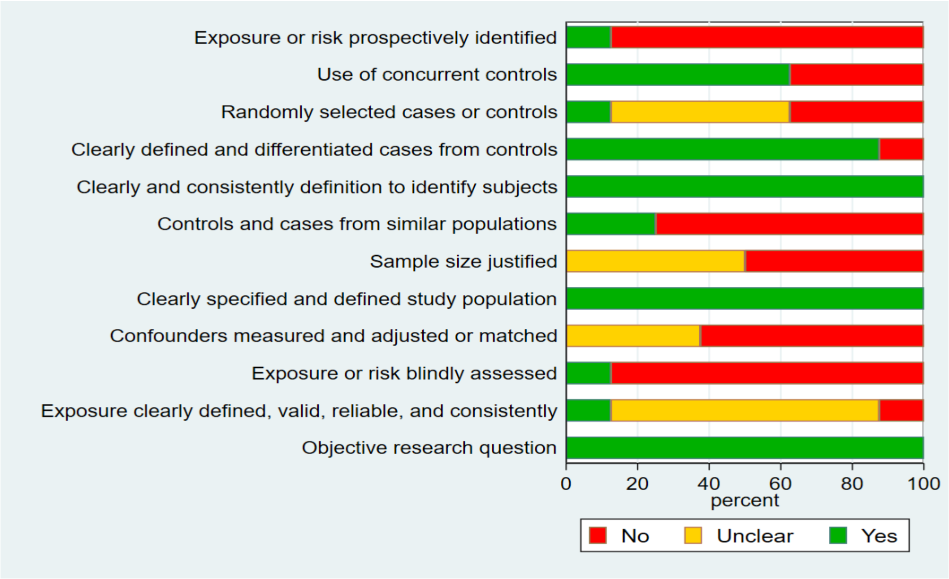

Table S1. Search Strategy to Identify Relevant Articles.

S/N	Concept	Search Terms
#1	Disease	("Coronavirus"[Mesh] OR coronavirus disease[tw] OR coronavirus[tw] OR 2019-nCov[tw] OR SARS-CoV-2[tw] OR COVID-19[tw] OR new coronary pneumonia[tw] OR corona virus[tw] OR novel coronavirus[tw] OR nCov[tw] OR SARS-2-CoV[tw] OR wuhan coronavirus[tw] OR wuhan pneumonia[tw])
#2	Clinical, Laboratory, Radiologic Characteristics, and Outcomes	(symptom*[tw] OR clinical characteristic*[tw] OR co-morbidit*[tw] OR comorbidit*[tw] OR epidemiolog*[tw] OR inciden*[tw] OR "Radiology"[Mesh] OR radiologic features[tw] OR chest x-ray[tw] OR chest CT[tw] OR Lung ultrasound[tw] OR "Ultrasonography"[Mesh] OR "Tomography, X-Ray Computed"[Mesh] OR ultrasound[tw] OR sonography[tw] OR ultrasonography[tw] OR radiography[tw] OR chest film[tw] OR chest radiograph[tw] risk factors[tw] OR "Diagnosis"[Mesh] OR cases[tw] OR "Mortality"[Mesh] OR death[tw] OR case fatality rate[tw] OR case fatality ratio[tw] OR "Physical Examination"[Mesh] OR "Clinical Laboratory Techniques"[Mesh] OR "Diagnostic Tests, Routine"[Mesh] OR clinical laboratory [tw] OR laboratory characteristics [tw] OR laboratory panel [tw] OR laboratory values [tw] OR "Critical Care"[Mesh] OR "Intensive Care Units"[Mesh] OR

Formatted Table

		Intensive Care Units[tw] OR "Hospitalization"[Mesh] OR "Sepsis"[Mesh])
#3	Publication period~~Species~~	("2019/12/31"[PDAT] : "2020/03/31"[PDAT])~~("humans"[Mesh])~~
#4	Publication type~~Publication period~~	(Review[ptyp] OR Practice Guideline[ptyp] OR Letter[ptyp] OR Guideline[ptyp] OR Editorial[ptyp] OR Comment[ptyp])~~("2019/12/31"[PDAT] : "2020/03/31"[PDAT])~~
#5	Species ~~Publication type~~	animals[mh] NOT humans[mh] ~~(Review[ptyp] OR Practice Guideline[ptyp] OR Letter[ptyp] OR Guideline[ptyp] OR Editorial[ptyp] OR Comment[ptyp])~~
#6~~Strategy~~	#1 AND #2 AND #3 NOT~~AND~~ #4 ~~NOT #5: Number of hits in PubMed = xxx (May 31, 2020)~~	
**Strategy**	#6 NOT #5~~: **Number of hits in PubMed** = xxx (March 31, 2020)~~	

Commented [A1]: from Cochrane
#10

Commented [A2R1]: I think it will technically give the same thing. But we can use that.

Commented [A3R1]:

#1	Systemic inflar
#2	Multiple (
#3	
#4	
#5	MicroRI
#6	
#7	
#8	

Commented [A4]: We might need to decide whether we need these filters or nit

Commented [A5]: May? March? Or April?

Commented [A6R5]: Sorry March, I have now corrected it

Formatted: Font: (Asian) Chinese (Taiwan)

Formatted: Font: Bold, (Asian) Chinese (Taiwan)

Table S2. Study characteristics of included studies.

Authors	Year	Country	Study Design	First date of enrollment	Number of observations	Average of age	Severity (%)	Mortality (%)	Male (%)	Cardiac comorbidity (%)	Hypertension (%)	Diabetes mellitus (%)	COPD (%)	Smoking (%)	Symptoms
Xia et al.	2020	China	Cohort	2020/1/23	20	NA	NA	NA	65.0	30.0	NA	NA	NA	NA	fever; cough; sore throat; fatigue; diarrhoea
Li et al.	2020	China	Cohort	2020/1/23	51	58	NA	NA	54.9	NA	NA	NA	NA	NA	fever; cough; fatigue
Zhu et al.	2020	China	Case control	2019/12/1	12	52	NA	NA	66.7	16.7	NA	NA	NA	NA	NA
Zhang et al.	2020	China	Cohort	2020/1/16	120	57	48.3	NA	59.2	10.0	35.0	14.2	1.7	7.5	fever; cough; fatigue; shortness of breath; nausea; diarrhoea
Yao et al.	2020	China	Cohort	2020/1/21	40	53.87	NA	NA	60.0	NA	NA	NA	NA	NA	fever; cough; sputum production; myalgia; fatigue; shortness of breath; headache; nausea; diarrhoea
Qin et al.	2020	China	Cohort	2020/1/10	452	58	63.3	NA	52.0	6.0	29.9	16.6	2.7	1.5	fever; cough; sputum production; sore throat; myalgia; fatigue; shortness of breath; headache; nausea; diarrhoea
Liu et al.	2020	China	Cohort	2020/1/15	56	57.5	NA	5.4	55.4	5.4	17.9	7.1	NA	39.3	fever; cough; sputum production; fatigue; shortness of breath
Chen et al.	2020	China	Cohort	2020/1/20	249	51	NA	0.8	50.6	22.1	NA	NA	NA	NA	fever; cough; sore throat; fatigue; shortness of breath; headache; diarrhoea
Bai et al.	2020	China	Case control	2020/1/6	219	44.8	NA	NA	54.3	5.5	14.2	8.2	4.1	NA	fever
Zhou et al.	2020	China	Cohort	2019/12/29	191	56	NA	28.3	62.3	7.9	30.4	18.8	3.1	5.8	fever; cough; sputum production; myalgia; fatigue; diarrhoea
Xu et al.	2020	China	Cohort	2020/1/23	90	50	NA	NA	43.3	3.3	18.9	5.6	1.1	NA	fever; cough; sputum production; sore throat; myalgia; fatigue; headache; nausea; diarrhoea
Xie et al.	2020	China	Case Series	NA	9	33	NA	NA	44.4	NA	NA	NA	NA	NA	fever; cough; fatigue; diarrhoea
Korea Centers	2020	Korea & China	Cohort	2020/1/20	28	42.6	NA	NA	53.6	NA	NA	NA	NA	NA	sore throat; myalgia; headache
Tian et al.	2020	China	Cohort	2020/1/20	262	47.5	17.6	1.2	48.5	NA	NA	NA	NA	NA	fever; cough; fatigue; shortness of breath; headache
Peng et al.	2020	China	Cohort	2020/1/20	112	62	14.3	15.2	47.3	55.4	82.1	20.5	NA	NA	fever; cough; shortness of breath; diarrhoea
Guan et al.	2020	China	Case Series	2020/1/22	7	50.37	NA	NA	42.9	NA	NA	NA	NA	NA	fever; cough; sputum production; sore throat; myalgia; fatigue; headache; diarrhoea
Zhao et al.	2020	China	Case control	2020/1/23	19	48	NA	NA	57.9	NA	10.5	NA	NA	NA	fever; cough; sore throat; fatigue; headache; diarrhoea
Qiu et al.	2020	China	Case Series	2020/2/2	8	26.142	NA	NA	50.0	NA	NA	NA	NA	NA	fever; cough; sore throat; headache; diarrhoea

Wu et al.	2020	China	Cohort	2020/1/21	40	45	NA	5.0	32.5	NA	NA	NA	NA	NA	fever; cough; sputum production; sore throat; myalgia; fatigue; headache; nausea; diarrhoea
Yang et al.	2020	China	Cohort	2020/1/17	149	45.11	NA	NA	54.4	NA	NA	NA	NA	NA	fever; cough; sputum production; sore throat; myalgia; shortness of breath; headache; nausea; diarrhoea
Chen et al.	2020	China	Cohort	2020/1/14	29	56	NA	6.9	72.4	NA	27.6	17.2	NA	6.9	fever; cough; sputum production; myalgia; fatigue; shortness of breath; headache; diarrhoea
Song et al.	2020	China	Cohort	2020/1/20	51	49	NA	NA	49.0	2.0	9.8	5.9	2.0	5.9	fever; cough; sputum production; sore throat; headache; nausea; diarrhoea
Huang et al.	2020	China	Cohort	2019/12/16	40	49	NA	15.0	75.0	15.0	15.0	20.0	2.5	7.5	fever; cough; sputum production; shortness of breath; headache; diarrhoea
Chung et al.	2020	China	Cohort	2020/1/18	21	51	NA	NA	61.9	NA	NA	NA	NA	NA	fever; cough; myalgia; fatigue; headache; nausea
Dai et al.	2020	China	Case Series	NA	4	52.5	NA	NA	100.0	NA	NA	NA	NA	NA	fever; cough; sputum production; sore throat; myalgia; headache; diarrhoea
Ruan et al.	2020	China	Cohort	NA	150	58.5	NA	45.3	68.0	8.7	34.7	16.7	2.0	NA	fever; cough; sputum production; myalgia; fatigue; shortness of breath
Liu et al.	2020	China	Cohort	2019/12/30	78	38	NA	2.6	50.0	NA	10.3	6.4	2.6	6.4	fever; cough
Zhao et al.	2020	China	Cohort	NA	101	44.44	NA	NA	55.5	NA	NA	NA	NA	NA	fever; cough; sore throat; shortness of breath; nausea; diarrhoea
Zhang et al.	2020	China	Cohort	2020/1/27	14	41	NA	NA	50.0	NA	NA	NA	NA	NA	fever; cough; diarrhoea
Young et al.	2020	Singapore	Case Series	2020/1/23	18	47	NA	NA	50.0	NA	NA	NA	NA	NA	fever; cough; sore throat; shortness of breath; diarrhoea
Cao et al.	2020	China	Case Series	2020/1/3	102	54	NA	NA	52.0	4.9	27.5	10.8	NA	NA	fever; cough; myalgia; fatigue; diarrhoea
Wang et al.	2020	China	Cohort	2020/1/25	31	7.08	NA	NA	48.4	NA	NA	NA	NA	NA	fever; cough; sputum production; sore throat; fatigue; shortness of breath; diarrhoea
Li et al.	2020	China	Cohort	2020/1/1	83	45.5	30.1	NA	53.0	1.2	6.0	8.4	6.0	NA	fever; cough; sputum production; sore throat; myalgia; shortness of breath; headache; diarrhoea
Huang et al.	2020	China	Cohort	2019/12/21	34	56.24	NA	NA	41.2	17.6	23.5	11.8	2.9	NA	fever; cough; sputum production; shortness of breath; headache; diarrhoea
Wu et al.	2020	China	Cohort	2020/1/22	80	46.1	NA	NA	48.8	NA	NA	NA	0.0	NA	fever; cough; sore throat; myalgia; shortness of breath; headache; nausea; diarrhoea
Guan et al.	2020	China	Cohort	2019/12/11	1081	47	16.0	2.8	58.9	2.5	15.3	7.5	1.1	14.6	fever; cough; sputum production; sore throat; shortness of breath; headache; diarrhoea

Cai et al.	2020	China	Case Series	2020/1/19	10	6.167	NA	NA	40.0	NA	NA	NA	NA	NA	fever; cough; sore throat; shortness of breath; diarrhoea
Liu et al.	2020	China	Cohort	2020/1/23	32	38.5	NA	NA	62.5	3.1	3.1	NA	NA	NA	fever; cough
Li et al.	2020	China	Case control	2020/1/1	31	54	NA	NA	48.4	NA	NA	NA	NA	NA	fever; cough; sputum production; sore throat; myalgia; fatigue; shortness of breath; headache; nausea; diarrhoea
Bernheim et al.	2020	China	Cohort	2020/1/18	121	45.3	NA	NA	50.4	NA	NA	NA	NA	NA	fever; cough; sputum production
Bai et al.	2020	China	Case Series	2020/1/26	6	49.5	NA	NA	NA	NA	NA	NA	NA	NA	fever; sore throat
Liu et al.	2020	China	Case Series	2020/1/11	12	53.67	NA	NA	66.7	33.3	25.0	16.7	NA	NA	fever; cough; myalgia; headache; diarrhoea
Kui et al.	2020	China	Cohort	2019/12/30	137	57	NA	11.7	44.5	7.3	9.5	10.2	1.5	NA	fever; cough; sputum production; shortness of breath; headache; diarrhoea
Ren et al.	2020	China	Case Series	2019/12/18	5	53.6	NA	20.0	60.0	NA	20.0	NA	NA	NA	fever; cough; sputum production; myalgia; fatigue; shortness of breath; headache; diarrhoea
Yang et al.	2020	China	Cohort	2019/12/24	52	59.7	NA	61.5	67.3	9.6	NA	17.3	NA	3.8	fever; cough; myalgia; fatigue; shortness of breath; headache
Wu et al.	2020	China	Cohort	2020/1/1	80	44	NA	NA	52.5	1.3	5.0	5.0	3.8	32.5	fever; cough; sputum production; sore throat; myalgia; shortness of breath; headache; diarrhoea
Shi et al.	2020	China	Cohort	2019/12/20	81	49.5	NA	3.7	51.9	9.9	14.8	12.3	NA	NA	fever; cough; sputum production; fatigue; shortness of breath; headache; diarrhoea
Bai et al.	2020	China	Case Series	2020/1/22	5	53.42	NA	20.0	60.0	20.0	20.0	40.0	NA	NA	fever; cough; fatigue
Wang et al.	2020	China	Case Series	2020/1/21	4	44.25	NA	NA	75.0	NA	NA	NA	NA	NA	fever; cough; fatigue; headache
Wang et al.	2020	China	Case Series	2020/1/1	138	56	NA	NA	54.4	14.5	31.2	10.1	2.9	NA	fever; cough; sputum production; sore throat; myalgia; fatigue; shortness of breath; headache; nausea; diarrhoea
Feng et al.	2020	China	Case Series	2020/1/16	15	7.86	NA	NA	33.3	NA	NA	NA	NA	NA	fever; cough; sore throat
Xie et al.	2020	China	Case Series	2020/1/16	5	48.4	NA	NA	100.0	NA	NA	NA	NA	NA	fever; cough; fatigue; shortness of breath
Xu et al.	2020	China	Case Series	2020/1/10	62	41	NA	NA	56.5	NA	8.1	1.6	1.6	NA	fever; cough; sputum production; headache; diarrhoea
Xiong et al.	2020	China	Cohort	2020/1/11	42	49.5	NA	NA	59.5	NA	NA	NA	NA	NA	fever; cough; fatigue; shortness of breath; diarrhoea
Zhou et al.	2020	China	Cohort	2020/1/16	62	52.8	NA	NA	62.9	NA	6.5	6.5	NA	NA	fever; cough; sputum production; myalgia; fatigue; shortness of breath
Xu et al.	2020	China	Case Series	2020/1/22	10	7.543	NA	NA	60.0	NA	NA	NA	NA	NA	fever; cough; sore throat; diarrhoea

Mo et al.	2020	China	Cohort	2020/1/1	155	54	NA	NA	55.5	NA	23.9	9.7	3.2	NA	fever; cough; fatigue; shortness of breath; headache; nausea; diarrhoea
Cheng et al.	2020	China	Case control	2020/1/19	11	50.36	NA	NA	72.7	NA	NA	NA	NA	NA	fever; cough; sputum production; sore throat; myalgia; shortness of breath; diarrhoea
Xu et al.	2020	China	Cohort	2020/1/23	51	41.67	NA	NA	98.0	NA	NA	7.8	NA	NA	fever; cough; sputum production; sore throat; myalgia; fatigue; shortness of breath; diarrhoea
Wang et al.	2020	China	Cohort	2020/1/16	69	42	NA	7.3	46.4	NA	13.0	10.1	5.8	NA	fever; cough; sputum production; sore throat; myalgia; fatigue; shortness of breath; headache; diarrhoea
Sun et al.	2020	China	Case Series	2020/1/24	8	6.81	NA	NA	75.0	NA	NA	NA	NA	NA	fever; cough; sputum production; myalgia; fatigue; headache; nausea; diarrhoea
Wang et al.	2020	China	Cohort	2020/1/11	55	49	NA	NA	40.0	NA	14.5	NA	NA	NA	fever; cough
Zhang et al.	2020	China	Case Series	2020/1/21	4	52	NA	NA	25.0	NA	NA	NA	NA	NA	fever; cough; sputum production; sore throat; myalgia; fatigue; shortness of breath; headache
Lin et al.	2020	China	Cohort	2020/1/20	92	41.5	NA	NA	NA	NA	NA	NA	NA	NA	fever; cough; shortness of breath
Liu et al.	2020	China	Cohort	2020/1/21	73	37.38	32.9	NA	56.2	NA	NA	NA	NA	NA	fever; cough; sputum production; fatigue
Liu et al.	2020	China	Case Series	2020/1/2	6	3.5	NA	NA	33.3	NA	NA	NA	NA	NA	fever; cough
Han et al.	2020	China	Cohort	2020/1/4	108	45	NA	NA	35.2	NA	NA	NA	NA	NA	fever; cough; sore throat; myalgia; fatigue; headache; diarrhoea
Ki et al.	2020	Korea	Case Series	2020/1/20	28	42	NA	NA	53.6	NA	NA	NA	NA	NA	fever; cough; sputum production; sore throat; myalgia; fatigue; headache
Cai et al.	2020	China	Cohort	2020/1/24	80	47	NA	NA	43.8	NA	NA	NA	NA	NA	fever; cough; sore throat; headache; diarrhoea
He et al.	2020	China	Cohort	2020/2/3	54	68	NA	48.2	63.0	14.8	44.4	24.1	3.7	NA	fever; cough; myalgia; shortness of breath; diarrhoea
Zhu et al.	2020	China	Case control	2020/1/24	32	40	NA	NA	46.9	15.6	68.8	31.3	18.8	NA	fever; cough; sputum production; headache; diarrhoea
Pung et al.	2020	Singapore	Cohort	NA	17	40	NA	NA	41.2	NA	5.9	NA	NA	NA	fever; cough; sore throat; myalgia; shortness of breath; diarrhoea
Cheng et al.	2020	China	Cohort	2020/1/28	615	63	NA	18.4	59.7	NA	37.9	16.3	2.1	NA	fever
Liu et al.	2020	China	Case Series	2020/1/22	10	42	NA	NA	40.0	NA	10.0	NA	NA	NA	fever; cough; sputum production; sore throat; headache; nausea
Lu et al.	2020	China	Cohort	2020/1/28	171	6.7	NA	0.6	60.8	NA	NA	NA	NA	NA	fever; cough; sore throat; fatigue; diarrhoea
Chen et al.	2020	China	Case Series	NA	9	42.11	NA	NA	55.6	NA	NA	NA	NA	NA	fever; cough; diarrhoea
Cao et al.	2020	China	Cohort	2020/1/18	199	58	NA	NA	60.3	NA	NA	11.6	NA	NA	fever

Wang et al.	2020	China	Cohort	2020/1/16	90	45	NA	2.2	36.7	NA	NA	NA	NA	NA	fever; cough; sore throat; myalgia; fatigue; headache; diarrhoea
Zhao et al.	2020	China	Cohort	2020/1/1	80	44	NA	NA	53.8	NA	NA	NA	NA	NA	fever
Yuan et al.	2020	China	Cohort	2020/1/1	27	60	NA	37.0	44.4	NA	18.5	22.2	NA	NA	fever; cough; myalgia; shortness of breath
Arentz et al.	2020	American	Cohort	2020/2/20	21	70	NA	52.4	52.4	52.4	NA	33.3	33.3	NA	fever; cough; shortness of breath
Qiu et al.	2020	China	Case Series	2020/2/4	10	65.7	NA	NA	0.0	NA	30.0	30.0	NA	NA	fever; cough
Chu et al.	2020	China	Cohort	2020/1/7	54	39	79.6	NA	66.7	NA	NA	NA	NA	NA	fever; cough; sputum production; sore throat; myalgia; fatigue; shortness of breath; nausea; diarrhoea
Qiang Ding et al.	2020	China	Case Series	NA	5	50.2	NA	NA	40.0	NA	NA	NA	NA	NA	fever; cough; sputum production; sore throat; myalgia; fatigue; shortness of breath; headache
Hu et al.	2020	China	Case Series	2020/1/28	24	32.5	NA	NA	33.3	NA	NA	NA	NA	NA	fever; cough; fatigue
Li et al.	2020	China	Case Series	2020/1/28	5	3.05	NA	NA	80.0	NA	NA	NA	NA	NA	fever; cough; sputum production; sore throat
Wang et al.	2020	China	Cohort	2020/1/7	60	60	NA	NA	36.7	NA	15.0	10.0	NA	NA	fever; cough; myalgia; shortness of breath
Wang et al.	2020	China	Cohort	2020/1/1	339	71	NA	19.2	49.0	NA	40.7	15.9	6.2	NA	fever; cough; sputum production; sore throat; myalgia; fatigue; shortness of breath; headache; nausea; diarrhoea
Xu et al.	2020	China	Cohort	2020/1/1	50	43.9	26.0	NA	58.0	NA	NA	NA	NA	NA	fever; cough; sputum production; sore throat; myalgia; fatigue; shortness of breath; headache
Zhang et al.	2020	China	Case Series	2020/1/18	9	35.22	NA	NA	55.6	NA	NA	11.1	NA	NA	fever; cough; sputum production; sore throat; myalgia; fatigue; diarrhoea
Zhou et al.	2020	China	Cohort	2020/1/28	17	41.84	29.4	NA	35.3	NA	NA	NA	NA	NA	fever; cough; fatigue; shortness of breath
Tolia et al.	2020	America	Cohort	2020/3/10	29	NA	NA	NA	55.2	NA	NA	NA	NA	NA	fever
Lo et al.	2020	China	Cohort	2020/1/21	10	54	NA	NA	30.0	NA	30.0	NA	NA	NA	fever; cough; sore throat; myalgia; shortness of breath; nausea; diarrhoea
Huang et al.	2020	China	Cohort	2020/1/22	25	46.9	NA	NA	56.0	NA	NA	NA	NA	NA	fever; cough; sputum production; sore throat; myalgia; fatigue
Gou et al.	2020	China	Cohort	NA	91	NA	NA	NA	42.9	NA	NA	NA	NA	NA	fever; cough; fatigue
Escalera-Antezana et al.	2020	Bolivia	Case Series	2020/3/2	12	36.5	NA	NA	50.0	NA	8.3	NA	NA	NA	fever; cough; sore throat; myalgia; fatigue; headache; diarrhoea
Cao et al.	2020	China	Cohort	2020/1/3	102	54	NA	16.7	52.0	NA	27.5	10.8	NA	NA	fever; cough; myalgia; fatigue; diarrhoea
Cai et al.	2020	China	Cohort	2020/1/11	298	47.5	19.5	1.0	48.7	NA	15.8	6.0	NA	NA	fever; cough; sore throat; fatigue; headache; diarrhoea

Jin et al.	2020	China	Cohort	2020/1/17	651	45.615	NA	NA	50.8	NA	15.4	7.4	0.2	NA	fever; cough; sputum production; sore throat; myalgia; fatigue; shortness of breath; headache; nausea; diarrhoea
Guo et al.	2020	China	Cohort	2020/1/23	187	58.5	NA	23.0	48.7	11.2	32.6	15.0	2.1	NA	NA
Guo et al.	2020	China	Cohort	2020/2/10	174	59	NA	5.2	43.7	NA	24.7	21.3	NA	NA	fever; cough; sore throat; myalgia; fatigue; shortness of breath; headache; nausea; diarrhoea
Chen et al.	2020	China	Case Series	2020/1/13	274	62	NA	NA	62.4	NA	33.9	17.2	NA	NA	fever; cough; sputum production; sore throat; myalgia; fatigue; shortness of breath; headache; nausea; diarrhoea
Bhatraju et al.	2020	USA	Case Series	2020/2/24	24	64	NA	50.0	62.5	NA	NA	58.3	4.2	NA	fever; cough; sputum production; sore throat; shortness of breath; headache
Chen et al.	2020	China	Cohort	2019/12/15	21	56	52.4	19.1	81.0	NA	23.8	14.3	NA	NA	fever; cough; sputum production; myalgia; fatigue; shortness of breath; headache; diarrhoea
Tang et al.	2020	China	Case control	2019/12/24	73	62	NA	NA	61.6	NA	95.9	47.9	NA	NA	fever; cough; sputum production; myalgia; fatigue; shortness of breath; nausea; diarrhoea
Yu et al.	2020	China	Cohort	2019/12/30	12	66	NA	25.0	83.3	NA	NA	NA	NA	NA	fever; cough; shortness of breath
Li et al.	2020	China	Cohort	2020/1/18	78	44.6	NA	NA	48.7	NA	12.8	5.1	11.5	NA	fever; cough; sputum production; sore throat; myalgia; fatigue; shortness of breath; headache; nausea; diarrhoea
Iwasawa et al.	2020	Japan	Case Series	2020/2/1	6	69	NA	NA	33.3	NA	16.7	16.7	NA	NA	fever; fatigue
Wang et al.	2020	China	Case Series	NA	5	57.6	NA	NA	60.0	NA	80.0	20.0	NA	NA	fever; cough; fatigue; shortness of breath; diarrhoea
Lescure et al.	2020	China	Case Series	2020/1/24	5	47	NA	20.0	60.0	NA	20.0	NA	NA	NA	fever; cough; shortness of breath; diarrhoea
Zheng et al.	2020	China	Cohort	2020/2/15	55	60.5	38.2	NA	43.6	NA	NA	NA	NA	NA	fever; cough
Zhang et al.	2020	China	Case Series	2020/2/5	7	59.85	NA	71.4	57.1	NA	42.9	28.6	NA	NA	fever; cough; shortness of breath; diarrhoea
Zhang et al.	2020	China	Cohort	2020/1/16	95	49	33.7	NA	55.8	NA	NA	NA	NA	NA	fever
Yuan et al.	2020	China	Cohort	2020/1/5	94	40	11.7	NA	44.7	6.4	9.6	5.3	NA	NA	fever; cough; sore throat; fatigue; diarrhoea
Xio et al.	2020	China	Cohort	2020/1/11	42	49.5	NA	NA	59.5	NA	NA	NA	NA	NA	fever; cough; fatigue; shortness of breath; diarrhoea
Wu et al.	2020	China	Cohort	2020/1/20	280	43.12	29.6	NA	53.9	NA	NA	NA	0.4	NA	fever; cough; sore throat; myalgia; shortness of breath; headache; nausea; diarrhoea
Wong et al.	2020	China	Cohort	2020/1/1	64	56	NA	NA	40.6	NA	20.3	12.5	NA	NA	fever; cough; sputum production; sore throat; shortness of breath; diarrhoea

Qiu et al.	2020	China	Cohort	2020/1/17	36	8.3	NA	NA	63.9	NA	NA	NA	NA	NA	fever; cough; sore throat; headache
Zheng et al.	2020	China	Cohort	2020/2/1	25	3	NA	NA	56.0	NA	NA	NA	NA	NA	fever; cough; shortness of breath; diarrhoea
Zhang et al.	2020	China	Cohort	2020/1/18	199	45.33	NA	NA	46.7	NA	50.3	24.1	0.5	NA	fever
Zhang et al.	2020	China	Cohort	2020/1/13	28	65	NA	28.6	60.7	NA	NA	14.3	NA	NA	fever; cough; myalgia; fatigue; shortness of breath; diarrhoea
Zhou et al.	2020	China	Cohort	2020/1/21	62	47.26	NA	NA	54.8	NA	NA	NA	NA	NA	fever
Liu et al.	2020	China	Case Series	NA	5	6.036	NA	NA	80.0	NA	NA	NA	NA	NA	fever; cough; diarrhoea
Wang et al.	2020	China	Cohort	2020/1/25	114	53	NA	NA	50.9	NA	28.9	NA	NA	NA	fever; cough; sputum production; sore throat; shortness of breath; diarrhoea
Xie et al.	2020	China	Cohort	2020/2/2	79	60	35.4	NA	55.7	8.9	17.7	10.1	NA	NA	fever; cough; sputum production; shortness of breath; diarrhoea
Wan et al.	2020	China	Cohort	2020/1/23	135	47	29.6	0.7	53.3	NA	9.6	8.9	NA	NA	fever; cough; sputum production; sore throat; shortness of breath; headache; nausea; diarrhoea
Lu et al.	2020	China	Case Series	NA	5	52.4	NA	NA	20.0	NA	NA	NA	NA	NA	fever; cough; sputum production; fatigue; shortness of breath
Dong et al.	2020	China	Case Series	NA	11	36.63	NA	NA	45.5	NA	9.1	NA	9.1	NA	fever; cough; sputum production; sore throat; myalgia; fatigue; shortness of breath; headache; nausea; diarrhoea
Albano et al.	2020	Italy	Case Series	2020/3/16	7	64.57	NA	NA	28.6	NA	NA	NA	NA	NA	fever; cough; sputum production; fatigue; shortness of breath
Zhu et al.	2020	China	Cohort	2020/1/18	72	55.6	NA	NA	58.3	NA	NA	NA	NA	NA	fever; cough; fatigue; shortness of breath
Yu et al.	2020	China	Case Series	2020/1/21	4	74.25	NA	25.0	100.0	NA	25.0	NA	25.0	NA	fever; cough
Pan et al.	2020	China	Cohort	2020/1/12	21	40	NA	NA	28.6	NA	NA	NA	NA	NA	fever; cough; sputum production; sore throat; myalgia; fatigue
Chen et al.	2020	China	Case Series	2020/1/1	99	55.5	NA	11.1	67.7	NA	NA	12.1	NA	NA	fever; cough; sore throat; myalgia; shortness of breath; headache; nausea; diarrhoea
Chen et al.	2020	China	Case control	2020/1/26	78	45	NA	NA	50.0	NA	NA	1.3	NA	NA	fever; cough; sputum production; sore throat; fatigue; shortness of breath; headache; diarrhoea
Diao et al.	2020	China	Case Series	2020/1/17	6	47.5	NA	NA	50.0	NA	NA	NA	NA	NA	fever; cough; diarrhoea
Z. F. Chen et al.	2020	China	Cohort	2020/1/20	98	43	NA	NA	53.1	NA	NA	NA	NA	NA	fever; cough; sore throat; shortness of breath
Chan JF et al.	2020	China	Case Series	2020/1/10	5	48	NA	NA	60.0	NA	40.0	20.0	NA	NA	fever; cough; sputum production; sore throat; diarrhoea
Luo S et al.	2020	China	Case Series	2020/1/1	183	53.8	NA	3.8	55.7	NA	NA	NA	NA	NA	nausea; diarrhoea
Wu C et al.	2020	China	Cohort	2019/12/25	201	51	41.8	21.9	63.7	NA	19.4	10.9	NA	NA	fever; cough; shortness of breath

Li et al.	2020	China	Cohort	2020/1/20	80	47.5	21.3	0.0	50.0	6.3	17.5	12.5	3.8	NA	fever; cough; sputum production; myalgia; fatigue; shortness of breath; headache; diarrhoea
Chen et al.	2020	China	Cohort	2020/1/27	296	NA	10.1	0.0	46.3	NA	NA	NA	NA	NA	NA
Jing Liu et al.	2020	China	Cohort	2020/1/5	40	48.7	NA	7.5	37.5	NA	15.0	15.0	NA	12.5	fever; cough; sputum production; myalgia; fatigue; shortness of breath; headache; nausea; diarrhoea
Yang et al.	2020	China	Cohort	2020/1/7	93	46.4	NA	0.0	60.2	14.0	24.7	22.6	NA	NA	fever; cough
Gang et al.	2020	United States	Cohort	2020/3/1	180	NA	NA	NA	NA	25.0	43.9	26.1	9.4	NA	cough; sore throat; myalgia; shortness of breath; headache; diarrhoea
X K et al.	2020	China	Cohort	2020/1/23	143	45.13	25.2	0.7	51.1	3.5	11.9	7.0	NA	NA	fever; cough; sore throat; myalgia; fatigue; shortness of breath; headache
Annemarie et al.	2020	British	Cohort	2020/2/6	20133	72.9	NA	25.7	59.9	27.2	NA	18.1	15.5	4.2	fever; cough; shortness of breath; nausea; diarrhoea
Giacomo et al.	2020	Italy	Cohort	2020/2/20	1591	63	NA	25.5	82.0	14.0	32.0	11.3	2.6	NA	NA
Safiya et al.	2020	America	Case Series	2020/3/1	5700	63	NA	9.7	60.3	16.9	53.1	31.7	5.0	9.8	fever
L YF et al.	2020	China	Cohort	NA	50	50.4	NA	NA	56.0	NA	16.0	6.0	NA	NA	fever; cough; sputum production; myalgia; fatigue; shortness of breath; headache; diarrhoea
W J et al.	2020	China	Cohort	2020/1/19	52	44	NA	NA	55.8	NA	NA	NA	NA	NA	fever; cough; myalgia; fatigue; headache
Li X et al.	2020	China	Case Series	2020/1/26	12	37	NA	NA	66.7	NA	16.7	NA	NA	33.3	fever; cough; myalgia; fatigue; headache; diarrhoea
Z FY et al.	2020	China	Cohort	NA	30	50.17	NA	NA	60.0	NA	NA	NA	NA	NA	fever; cough
Z YJ et al.	2020	China	Cohort	20-Jan	26	39.77	NA	NA	69.2	NA	15.4	11.5	NA	NA	fever; cough; sputum production; sore throat; myalgia; fatigue; shortness of breath; headache; diarrhoea
Sun HY et al.	2020	China	Cohort	2020/1/21	88	48.52	NA	NA	55.7	6.8	25.0	11.4	NA	NA	fever; cough; sputum production; sore throat; myalgia; headache
Cheng DZ et al.	2020	China	Cohort	2020/1/1	54	60.1	NA	NA	53.7	13.0	38.9	18.5	NA	NA	fever; cough; fatigue; shortness of breath
Xiang TX et al.	2020	China	Cohort	2020/1/21	49	42.9	18.4	NA	67.4	NA	12.2	4.1	NA	6.1	fever; cough; sputum production; sore throat; fatigue; diarrhoea
Xiong J et al.	2020	China	Cohort	2020/1/17	89	53	34.8	7.9	46.1	NA	29.2	15.7	5.6	NA	fever; cough; sputum production; sore throat; myalgia; fatigue; shortness of breath; nausea; diarrhoea
Fang L et al.	2020	China	Cohort	2020/1/25	308	NA	NA	5.2	49.4	NA	NA	NA	NA	NA	fever; cough; fatigue; diarrhoea
Cheng KB et al.	2020	China	Cohort	2020/1/1	463	51	39.1	NA	52.7	6.0	23.1	8.6	4.1	4.5	fever; cough; sputum production; sore throat; myalgia; fatigue; shortness of breath; headache; diarrhoea

Chen T et al.	2020	China	Cohort	NA	76	59.5	NA	4.0	52.6	6.6	30.3	17.1	NA	NA	fever; cough; sore throat; myalgia; fatigue; nausea
Cheng JL et al.	2020	China	Cohort	NA	1079	46	NA	1.0	53.1	NA	NA	NA	NA	NA	fever; cough; sputum production; sore throat; fatigue; shortness of breath; headache; nausea; diarrhoea
Xu S et al.	2020	China	Cohort	2020/1/8	62	62.9	NA	22.6	62.9	17.7	41.9	14.5	8.1	NA	fever; cough; sputum production; sore throat; myalgia; fatigue; shortness of breath; nausea; diarrhoea
Sun WW et al.	2020	China	Cohort	NA	337	NA	NA	NA	47.8	NA	NA	NA	NA	NA	fever; cough; sore throat; myalgia; fatigue; diarrhoea
SM Y et al.	2020	China	Cohort	2020/1/21	40	39.9	NA	NA	65.0	NA	NA	NA	NA	NA	fever; cough; sore throat; myalgia; fatigue; diarrhoea
COVID-19 National Incident Room Surveillance T	2020	Australia	Cohort	NA	71	NA	NA	2.8	NA	NA	NA	NA	NA	NA	fever; cough; sore throat; myalgia; shortness of breath; headache; nausea; diarrhoea
Lin M et al.	2020	Canadian	Cohort	2020/1/20	135	28	NA	NA	43.7	NA	NA	NA	NA	NA	fever; sore throat; fatigue
Du W et al.	2020	China	Cohort	2020/1/23	67	34.1	NA	NA	47.8	NA	NA	NA	NA	NA	fever; cough; sputum production; myalgia; fatigue; shortness of breath; headache; nausea; diarrhoea
Ma H et al.	2020	China	Cohort	2020/1/21	158	NA	NA	0.0	26.6	5.1	NA	NA	NA	NA	fever; cough; sore throat; diarrhoea
Li H et al.	2020	China	Cohort	2020/1/24	40	5.09	NA	NA	57.5	NA	NA	NA	NA	NA	fever; cough; sore throat; fatigue; diarrhoea
Song W et al.	2020	China	Case Series	2020/1/1	16	8.5	NA	NA	62.5	NA	NA	NA	NA	NA	fever; cough; sore throat
D YH et al.	2020	China	Case Series	2020/1/27	7	40	NA	NA	57.1	NA	NA	NA	NA	NA	fever; cough
Yaoling M et al.	2020	China	Cohort	NA	115	NA	NA	NA	63.5	NA	NA	NA	NA	NA	fever
Zhang B et al.	2020	China	Cohort	2020/1/20	46	8.75	NA	NA	63.0	NA	NA	NA	NA	NA	fever; cough
Xin T et al.	2020	China	Case Series	2020/1/17	13	7.90769	NA	NA	30.8	NA	NA	NA	NA	NA	fever; cough; diarrhoea
Wu Q et al.	2020	China	Cohort	2020/1/20	74	6	NA	0.0	59.5	NA	NA	NA	NA	NA	fever; cough; sputum production; myalgia; shortness of breath; headache; diarrhoea
Li B et al.	2020	China	Cohort	2020/1/16	22	8	NA	NA	54.6	NA	NA	NA	NA	NA	fever; cough
Parri N et al.	2020	Italy	Cohort	2020/3/3	100	3.3	NA	0.0	57.0	NA	NA	NA	NA	NA	fever; cough; sore throat; fatigue; shortness of breath; headache; diarrhoea
Garazzino S et al.	2020	Italy	Cohort	2020/4/10	168	5	NA	NA	56.0	NA	NA	NA	NA	NA	fever; cough; fatigue; shortness of breath; diarrhoea

COVID-19 National Emergency Response Center	2020	Korea	Cohort	2020/1/20	7755	77	NA	0.9	0.5	0.1	0.4	0.3	NA	NA	NA
Chang D et al.	2020	China	Case Series	2020/1/16	13	34	NA	NA	76.9	NA	NA	NA	NA	NA	fever; cough; sputum production; myalgia; headache; diarrhoea
Tan YP et al.	2020	China	Case Series	2020/1/27	10	7	NA	0.0	30.0	NA	NA	NA	NA	NA	fever; cough; sore throat
Cao et al.	2020	China	Cohort	2020/1/1	128	NA	16.4	NA	46.9	NA	NA	NA	NA	NA	fever; cough; sore throat
Feng et al.	2020	China	Cohort	2020/1/1	476	50	NA	8.0	56.9	8.0	23.7	10.3	4.6	9.2	fever; cough; sputum production; myalgia; shortness of breath
G L et al.	2020	China	Case Series	2020/1/1	10	41.8	NA	NA	60.0	NA	NA	NA	NA	NA	fever; cough; sore throat
Zheng et al.	2020	China	Cohort	2020/1/17	161	45	18.6	NA	49.7	2.5	13.7	4.3	3.7	NA	fever; cough; myalgia; fatigue; shortness of breath; headache; diarrhoea
Lian J et al.	2020	China	Cohort	2020/1/17	788	45.83	NA	NA	51.8	1.4	16.0	7.2	0.4	6.9	fever; cough; sputum production; sore throat; fatigue; shortness of breath; headache
Shi S et al.	2020	China	Cohort	2020/1/20	416	64	NA	13.7	49.3	10.6	30.5	14.4	2.9	NA	fever; cough; sputum production; sore throat; fatigue; shortness of breath; headache; diarrhoea
Yang S et al.	2020	China	Cohort	2020/1/20	44	48.5	NA	NA	56.8	NA	NA	NA	NA	NA	fever; cough; fatigue
Deng Y et al.	2020	China	Cohort	2020/1/1	225	NA	NA	11.1	55.1	7.6	25.8	11.6	NA	NA	fever; cough; sputum production; shortness of breath; headache; diarrhoea

Table S3. Meta-analysis of the symptoms

Symptoms	Group	Number of Studies	Proportion (95% C.I.)	Q	I^2	*p*-value
**Mortality**	**Overall**	61	0.10 (0.06-0.14)	6342.74	99.1%	
	**Study Design**					
	Cohort	51	0.09 (0.06-0.13)	6213.29	99.2%	*0.101*
	Case Series	10	0.17 (0.05-0.33)	49.49	81.8%	
	Case Control	0	NA	NA	NA	
	**Age of participant**					
	Adult	52	0.12 (0.08-0.17)	5966.21	99.1%	***<0.0001***
	Children	4	0.00 (0.00-0.005)	0.80	0.0%	
	**Ethnics**					
	China	53	0.09 (0.06-0.13)	1118.57	95.4%	*0.462*
	Non-China	8	0.15 (0.03-0.32)	4668.46	99.9%	
**Severity**	**Overall**	27	0.31 (0.25-0.37)	548.58	95.3%	
	**Study Design**					
	Cohort	27	0.31 (0.25-0.37)	548.58	95.3%	NA
	Case Series	NA	NA	NA	NA	
	Case Control	NA	NA	NA	NA	
	**Age of participant**					
	Adult	25	0.31 (0.25-0.38)	537.07	95.5%	NA
	Children	NA	NA	NA	NA	
	**Ethics**					
	China	27	0.31 (0.25-0.37)	548.58	95.3%	NA
	Non-China	NA	NA	NA	NA	
**Fever**	**Overall**	182	0.76 (0.72-0.79)	7618.08	97.6%	
	**Study Design**					
	Cohort	123	0.76 (0.73-0.79)	3645.58	96.7%	*0.256*
	Case Series	52	0.73 (0.64-0.81)	1663.15	96.9%	
	Case Control	7	0.82 (0.72-0.91)	56.11	89.3%	
	**Age of participant**					

	Adult	152	0.80 (0.77-0.83)	7068.88	97.9%	***<0.0001***
	Children	20	0.52 (0.41-0.62)	145.16	86.9%	
	**Ethics**					
	China	167	0.78 (0.74-0.81)	3951.68	95.8%	***<0.0001***
	Non-China	15	0.53 (0.40-0.65)	1984.65	99.3%	
**Cough**	**Overall**	169	0.56 (0.52-0.59)	3778.37	95.6%	
	**Study Design**					
	Cohort	115	0.54 (0.50-0.58)	3548.34	96.8%	***0.006***
	Case Series	48	0.61 (0.52-0.69)	193.48	75.7%	
	Case Control	6	0.72 (0.61-0.82)	9.72	48.5%	
	**Age of participant**					
	Adult	140	0.57 (0.53-0.61)	3071.04	95.5%	***0.018***
	Children	20	0.44 (0.34-0.54)	57.80	67.1%	
	**Ethics**					
	China	157	0.55 (0.52-0.59)	3246.54	95.2%	*0.343*
	Non-China	12	0.62 (0.49-0.74)	138.82	92.1%	
**Sputum production**	**Overall**	75	0.24 (0.21-0.29)	1486.46	95.0%	
	**Study Design**					
	Cohort	51	0.23 (0.19-0.28)	1366.32	96.3%	*0.108*
	Case Series	19	0.27 (0.18-0.37)	47.42	62.0%	
	Case Control	5	0.37 (0.23-0.52)	17.24	76.8%	
	**Age of participant**					
	Adult	70	0.25 (0.21-0.29)	1453.37	95.3%	*0.514*
	Children	4	0.16 (0.01-0.41)	16.57	81.9%	
	**Ethics**					
	China	72	0.25 (0.21-0.29)	1473.56	95.2%	*0.817*
	Non-China	3	0.21 (0.02-0.50)	12.62	84.2%	
**Sore throat**	**Overall**	85	0.12 (0.09-0.14)	781.72	89.3%	
	**Study Design**					
	Cohort	58	0.10 (0.08-0.13)	667.92	91.5%	***0.003***
	Case Series	23	0.20 (0.13-0.29)	96.84	77.3%	
	Case Control	4	0.12 (0.04-0.24)	8.38	64.2%	

	**Age of participant**					
	Adult	69	0.11 (0.08-0.14)	557.09	87.8%	*0.228*
	Children	10	0.17 (0.07-0.31)	108.14	91.7%	
	**Ethics**					
	China	76	0.10 (0.08-0.13)	660.39	88.6%	***0.011***
	Non-China	9	0.25 (0.14-0.38)	58.69	86.4%	
**Myalgia**	**Overall**	75	0.19 (0.16-0.23)	575.81	87.1%	
	**Study Design**					
	Cohort	55	0.18 (0.15-0.21)	466.29	88.4%	***0.0002***
	Case Series	17	0.27 (0.19-0.35)	40.61	60.6%	
	Case Control	3	0.35 (0.26-0.46)	0.83	0.0%	
	**Age of participant**					
	Adult	70	0.20 (0.17-0.24)	460.93	85.0%	*0.091*
	Children	2	0.01 (0.00-0.23)	3.90	74.4%	
	**Ethics**					
	China	70	0.19 (0.16-0.23)	533.54	87.1%	*0.584*
	Non-China	5	0.23 (0.11-0.37)	25.01	84.0%	
**Fatigue**	**Overall**	94	0.29 (0.25-0.33)	2157.66	95.7%	
	**Study Design**					
	Cohort	68	0.28 (0.23-0.33)	1806.42	96.3%	*0.203*
	Case Series	22	0.35 (0.25-0.44)	91.01	76.9%	
	Case Control	4	0.38 (0.14-0.66)	41.30	92.7%	
	**Age of participant**					
	Adult	84	0.31 (0.27-0.35)	1681.59	95.1%	***<0.0001***
	Children	6	0.06 (0.03-0.10)	12.96	61.4%	
	**Ethics**					
	China	87	0.30 (0.26-0.35)	2037.29	95.8%	***0.004***
	Non-China	7	0.12 (0.04-0.23)	35.59	83.1%	
**Shortness of breath**	**Overall**	95	0.24 (0.20-0.29)	8293.53	98.9%	
	**Study Design**					
	Cohort	75	0.22 (0.18-0.27)	8177.59	99.1%	*0.084*
	Case Series	16	0.36 (0.20-0.53)	74.37	79.8%	

	Case Control	4	0.39 (0.16-0.65)	30.78	90.3%	
	**Age of participant**					
	Adult	86	0.25 (0.21-0.30)	7769.77	98.9%	***<0.0001***
	Children	6	0.05 (0.01-0.10)	10.82	53.8%	
	**Ethics**					
	China	85	0.23 (0.18-0.27)	2498.05	96.6%	*0.202*
	Non-China	10	0.38 (0.16-0.62)	535.92	98.3%	
**Headache**	**Overall**	76	0.10 (0.08-0.12)	465.78	83.9%	
	**Study Design**					
	Cohort	53	0.09 (0.08-0.11)	399.19	87.0%	*0.071*
	Case Series	19	0.13 (0.07-0.19)	42.01	57.2%	
	Case Control	4	0.13 (0.05-0.24)	7.19	58.3%	
	**Age of participant**					
	Adult	69	0.10 (0.08-0.12)	441.14	84.6%	***0.048***
	Children	4	0.04 (0.005-0.09)	2.93	0.0%	
	**Ethics**					
	China	70	0.10 (0.08-0.12)	446.60	84.5%	*0.99*
	Non-China	6	0.10 (0.05-0.16)	14.88	66.4%	
**Nausea**	**Overall**	36	0.09 (0.05-0.14)	2176.27	98.4%	
	**Study Design**					
	Cohort	27	0.06 (0.04-0.09)	1845.77	98.6%	***0.008***
	Case Series	7	0.21 (0.04-0.45)	321.15	98.1%	
	Case Control	2	0.27 (0.10-0.48)	4.60	78.3%	
	**Age of participant**					
	Adult	34	0.09 (0.05-0.13)	2148.13	98.5%	***0.007***
	Children	1	0.5 (0.15-0.85)	0.00	NA	
	**Ethics**					
	China	34	0.09 (0.05-0.14)	715.09	95.4%	*0.767*
	Non-China	2	0.13 (0.00-0.43)	36.32	97.2%	
**Diarrhoea**	**Overall**	110	0.10 (0.08-0.12)	2885.30	96.2%	
	**Study Design**					
	Cohort	75	0.09 (0.07-0.11)	2728.63	97.3%	***0.003***

	Case Series	29	0.16 (0.10-0.22)	124.58	77.5%	
	Case Control	6	0.11 (0.04-0.22)	29.38	83.0%	
	**Age of participant**					
	Adult	91	0.10 (0.07-0.12)	2648.50	96.6%	*0.946*
	Children	12	0.09 (0.04-0.15)	16.57	33.6%	
	**Ethics**					
	China	102	0.09 (0.07-0.11)	860.88	88.3%	***0.004***
	Non-China	8	0.18 (0.13-0.24)	49.87	86.0%	

Table S4. Sensitivity analysis of outlier's analysis and publication bias.

Symptoms	Number of Studies	Pooled proportion before remove outlier	Number of removed Studies	Pooled proportion after remove outlier	Publication bias (Egger's test)
**Mortality**	61	0.10 (0.06-0.14)	31	0.09 (0.07-0.12)	p=0.145
**Fever**	182	0.76 (0.72-0.79)	82	0.78 (0.76-0.80)	p<0.05
**Cough**	169	0.56 (0.52-0.59)	68	0.55 (0.52-0.58)	p<0.05
**Sputum production**	75	0.24 (0.21-0.29)	25	0.24 (0.21-0.27)	p=0.270
**Sore throat**	85	0.12 (0.09-0.14)	23	0.11 (0.09-0.13)	p<0.05
**Myalgia**	75	0.19 (0.16-0.23)	22	0.20 (0.17-0.22)	p<0.05
**Fatigue**	94	0.29 (0.25-0.33)	38	0.29 (0.26-0.32)	p<0.05
**Shortness of breath**	95	0.24 (0.20-0.29)	49	0.24 (0.21-0.27)	p<0.05
**Headache**	76	0.10 (0.08-0.12)	12	0.10 (0.08-0.11)	p<0.05
**Nausea**	36	0.09 (0.05-0.14)	12	0.07 (0.05-0.10)	p<0.05
**Diarrhoea**	110	0.10 (0.08-0.12)	26	0.09 (0.08-0.11)	p<0.05

Table S5. Sensitivity analysis of different transformation methods.

Symptoms	Number of Studies	Raw proportion (PR)	Logit transformed (PLO)	Arcsine transformation (PAS)	Freeman-Tukey double arcsine transform (PFT)	GLMM
**Mortality**	61	0.13 (0.09-0.17)	0.08 (0.06-0.12)	0.10 (0.07-0.14)	0.10 (0.06-0.14)	0.07 (0.04-0.10)
**Fever**	182	0.74 (0.71-0.77)	0.76 (0.73-0.79)	0.76 (0.73-0.79)	0.76 (0.72-0.79)	0.77 (0.74-0.80)
**Cough**	169	0.56 (0.53-0.59)	0.56 (0.52-0.59)	0.56 (0.52-0.60)	0.56 (0.52-0.59)	0.56 (0.52-0.60)
**Sputum production**	75	0.26 (0.22-0.30)	0.24 (0.20-0.29)	0.25 (0.21-0.29)	0.24 (0.21-0.29)	0.23 (0.19-0.28)
**Sore throat**	85	0.13 (0.10-0.15)	0.12 (0.10-0.15)	0.12 (0.10-0.15)	0.12 (0.09-0.14)	0.11 (0.09-0.14)
**Myalgia**	75	0.21 (0.17-0.24)	0.20 (0.17-0.23)	0.20 (0.17-0.23)	0.19 (0.16-0.23)	0.19 (0.16-0.22)
**Fatigue**	94	0.30 (0.26-0.34)	0.28 (0.24-0.33)	0.29 (0.25-0.33)	0.29 (0.25-0.33)	0.27 (0.23-0.32)
**Shortness of breath**	95	0.27 (0.22-0.31)	0.23 (0.18-0.28)	0.25 (0.20-0.29)	0.24 (0.20-0.29)	0.22 (0.17-0.27)
**Headache**	76	0.10 (0.09-0.12)	0.10 (0.09-0.12)	0.10 (0.09-0.12)	0.10 (0.08-0.12)	0.10 (0.08-0.12)
**Nausea**	36	0.11 (0.06-0.16)	0.08 (0.05-0.12)	0.09 (0.06-0.14)	0.09 (0.05-0.14)	0.07 (0.05-0.11)
**Diarrhoea**	110	0.11 (0.09-0.13)	0.10 (0.08-0.12)	0.10 (0.08-0.12)	0.10 (0.08-0.12)	0.09 (0.08-0.11)

Table S6. Subgroup analysis for cohort study quality assessment.

Symptoms	Subgroup	Number of Studies	Proportion (95% C.I.)	Q	I^2	*p*-value
**Fever**	**Q6. Prospectively measured exposure**					
	Yes	5	0.46 (0.22-0.71)	414.78	99.0%	**0.015**
	No	65	0.77 (0.73-0.81)	1273.35	95.0%	
**Cough**	**Q6. Prospectively measured exposure**					
	Yes	3	0.59 (0.44-0.73)	26.34	92.4%	**0.351**
	No	62	0.51 (0.46-0.57)	2338.47	97.4%	
**Sputum production**	**Q6. Prospectively measured exposure**					
	Yes	0	NA	NA	NA	**NA**
	No	31	0.20 (0.14-0.26)	1044.85	97.1%	
**Sore throat**	**Q6. Prospectively measured exposure**					
	Yes	2	0.08 (0.01-0.21)	4.48	77.7%	**0.715**
	No	35	0.10 (0.08-0.13)	244.29	86.1%	
**Myalgia**	**Q6. Prospectively measured exposure**					
	Yes	1	0.30 (0.17-0.45)	0.00	NA	**0.064**
	No	30	0.17 (0.12-0.22)	315.01	90.8%	
**Fatigue**	**Q6. Prospectively measured exposure**					
	Yes	2	0.19 (0.02-0.45)	10.36	90.3%	**0.564**
	No	42	0.26 (0.20-0.32)	1352.84	97.0%	
**Shortness of breath**	**Q6. Prospectively measured exposure**					
	Yes	2	0.39 (0.00-0.94)	174.47	99.4%	**0.488**
	No	12	0.18 (0.07-0.32)	1130.08	99.0%	
**Headache**	**Q6. Prospectively measured exposure**					
	Yes	1	0.04 (0.01-0.09)	0.00	NA	**0.020**
	No	31	0.10 (0.08-0.13)	275.96	89.1%	
**Nausea**	**Q6. Prospectively measured exposure**					
	Yes	0	NA	NA	NA	**NA**
	No	15	0.06 (0.03-0.10)	53.49	73.8%	
**Diarrhoea**	**Q6. Prospectively measured exposure**					
	Yes	2	0.08 (0.03-0.13)	0.46	0.0%	**0.487**
	No	40	0.10 (0.07-0.13)	398.90	90.2%	

Table S7. Subgroup analysis for cohort study quality assessment.

Symptoms	Subgroup	Number of Studies	Proportion (95% C.I.)	Q	I^2	*p*-value
**Fever**	**Q8. Use of concurrent controls**					
	Yes	4	0.81 (0.72-0.89)	0.82	0.0%	**0.0087**
	No	2	0.61 (0.49-0.72)	2.16	53.6%	
**Cough**	**Q8. Use of concurrent controls**					
	Yes	4	0.66 (0.51-0.80)	5.85	48.7%	**0.86**
	No	1	0.68 (0.52-0.81)	0.00	NA	
**Sputum production**	**Q8. Use of concurrent controls**					
	Yes	0	NA	NA	NA	**NA**
	No	3	0.31 (0.12-0.54)	9.34	78.6%	
**Sore throat**	**Q8. Use of concurrent controls**					
	Yes	3	0.19 (0.09-0.31)	0.79	0.0%	**0.0304**
	No	1	0.05 (0.0009-0.14)	0.00	NA	
**Myalgia**	**Q8. Use of concurrent controls**					
	Yes	2	0.37 (0.21-0.55)	0.64	0.0%	**NA**
	No	0	NA	NA	NA	
**Fatigue**	**Q8. Use of concurrent controls**					
	Yes	2	0.34 (0.001-0.84)	13.84	92.8%	**0.278**
	No	1	0.10 (0.02-0.22)	0.00	NA	
**Shortness of breath**	**Q8. Use of concurrent controls**					
	Yes	1	0.09 (0.00-0.35)	NA	NA	**NA**
	No	0	NA	NA	NA	
**Headache**	**Q8. Use of concurrent controls**					
	Yes	3	0.12 (0.02-0.28)	6.96	71.2%	**NA**
	No	0	NA	NA	NA	
**Nausea**	**Q8. Use of concurrent controls**					
	Yes	1	0.16 (0.05-0.31)	NA	NA	**NA**
	No	0	NA	NA	NA	
**Diarrhoea**	**Q8. Use of concurrent controls**					
	Yes	4	0.06 (0.01-0.13)	1.27	0.0%	**0.708**
	No	1	0.05 (0.0009-0.14)	0.00	NA	

Table S8. Study quality assessment tools**.**

Study Design	Criteria	Yes	No	Unclear
**Cohort Study**	1. Was the research question or objective in this paper clearly stated?			
	2. Was the study population clearly specified and defined?			
	3. Were all the subjects selected or recruited from the same or similar populations (including the same time period)? Were inclusion and exclusion criteria for being in the study pre-specified and applied uniformly to all participants?			
	4. Was a sample size justification, power description, or variance and effect estimates provided?			
	5. For the analyses in this paper, were the exposure(s) of interest measured prior to the outcome(s) being measured?			
	6. Was the timeframe sufficient so that one could reasonably expect to see an association between exposure and outcome if it existed?			
	7. Were the outcome measures (dependent variables) clearly defined, valid, reliable, and implemented consistently across all study participants?			
	8. Were the outcome assessors blinded to the exposure status of participants?			
	9. Was loss to follow-up after baseline 20% or less?			
	10. Were key potential confounding variables measured and adjusted statistically for their impact on the relationship between exposure(s) and outcome(s)?			
**Case Series Study**	1. Was the study question or objective clearly stated?			
	2. Was the study population clearly and fully described, including a case definition?			
	3. Were the cases consecutive?			
	4. Were the subjects comparable?			
	5. Was the intervention clearly described?			
	6. Were the outcome measures clearly defined, valid, reliable, and implemented consistently across all study participants?			
	7. Was the length of follow-up adequate?			
	8. Were the statistical methods well-described?			
	9. Were the results well-described?			
**Case Control Study**	1. Was the research question or objective in this paper clearly stated and appropriate?			
	2. Was the study population clearly specified and defined?			
	3. Did the authors include a sample size justification?			
	4. Were controls selected or recruited from the same or similar population that gave rise to the cases (including the same timeframe)?			
	5. Were the definitions, inclusion and exclusion criteria, algorithms or processes used to identify or select cases and controls valid, reliable, and implemented consistently across all study participants?			

	6. Were the cases clearly defined and differentiated from controls?			
	7. If less than 100 percent of eligible cases and/or controls were selected for the study, were the cases and/or controls randomly selected from those eligible?			
	8. Was there use of concurrent controls?			
	9. Were the investigators able to confirm that the exposure/risk occurred prior to the development of the condition or event that defined a participant as a case?			
	10. Were the assessors of exposure/risk blinded to the case or control status of participants?			
	11. Were key potential confounding variables measured and adjusted statistically in the analyses? If matching was used, did the investigators account for matching during study analysis?			
